# Prenatal exposure to maternal smoking and offspring DNA methylation across the lifecourse: findings from the Avon Longitudinal Study of Parents and Children (ALSPAC)

**DOI:** 10.1093/hmg/ddu739

**Published:** 2014-12-30

**Authors:** Rebecca C. Richmond, Andrew J. Simpkin, Geoff Woodward, Tom R. Gaunt, Oliver Lyttleton, Wendy L. McArdle, Susan M. Ring, Andrew D.A.C. Smith, Nicholas J. Timpson, Kate Tilling, George Davey Smith, Caroline L. Relton

**Affiliations:** 1 MRC Integrative Epidemiology Unit (IEU), School of Social and Community Medicine, University of Bristol, Bristol BS8 2BN, UK and; 2 Institute of Genetic Medicine, Newcastle University, Newcastle upon Tyne NE1 3BZ, UK

## Abstract

Maternal smoking during pregnancy has been found to influence newborn DNA methylation in genes involved in fundamental developmental processes. It is pertinent to understand the degree to which the offspring methylome is sensitive to the intensity and duration of prenatal smoking. An investigation of the persistence of offspring methylation associated with maternal smoking and the relative roles of the intrauterine and postnatal environment is also warranted. In the Avon Longitudinal Study of Parents and Children, we investigated associations between prenatal exposure to maternal smoking and offspring DNA methylation at multiple time points in approximately 800 mother–offspring pairs. In cord blood, methylation at 15 CpG sites in seven gene regions (*AHRR*, *MYO1G*, *GFI1*, *CYP1A1*, *CNTNAP2*, *KLF13* and *ATP9A*) was associated with maternal smoking, and a dose-dependent response was observed in relation to smoking duration and intensity. Longitudinal analysis of blood DNA methylation in serial samples at birth, age 7 and 17 years demonstrated that some CpG sites showed reversibility of methylation (*GFI1*, *KLF13* and *ATP9A*), whereas others showed persistently perturbed patterns (*AHRR*, *MYO1G*, *CYP1A1* and *CNTNAP2*). Of those showing persistence, we explored the effect of postnatal smoke exposure and found that the major contribution to altered methylation was attributed to a critical window of *in utero* exposure. A comparison of paternal and maternal smoking and offspring methylation showed consistently stronger maternal associations, providing further evidence for causal intrauterine mechanisms. These findings emphasize the sensitivity of the methylome to maternal smoking during early development and the long-term impact of such exposure.

## Introduction

Despite the known health risks to both mothers and newborns, maternal smoking during pregnancy remains a significant public health problem in high-income countries and recent reports suggest that ∼12% of mothers in England are still smoking at the time of delivery (1). Exposure of the fetus to maternal smoking *in utero* has been associated with adverse perinatal outcomes, including low birth weight (2–4), elevated blood pressure (5,6), obesity (7,8) and behavioural difficulties in childhood (9,10). It has been proposed that epigenetic modifications such as DNA methylation may mediate the adverse developmental consequences associated with smoking during pregnancy (11).

Cigarette smoke is an established environmental associate of DNA methylation (12–17) and maternal smoking in pregnancy has recently been found to be associated with levels of DNA methylation in large-scale epigenome wide association studies (EWAS) of cord blood (18) and infant whole blood shortly after delivery (19). Of particular importance is the observation that maternal smoking during pregnancy is associated with changes in methylation in genes involved in fundamental developmental processes (18,19).

The associations found between maternal cotinine levels, an objective biomarker of smoking and DNA methylation in newborns imply a dose-dependent effect of maternal smoking in pregnancy (18). The sensitivity of the offspring methylome to the intensity (19) and duration (20) of smoking during pregnancy has been further explored. Of potential relevance is the impact of maternal smoking in early pregnancy, when women may not be aware that they are pregnant. During the early phases of embryogenesis, the products of tobacco smoke may induce soma-wide modification of DNA methylation in the exposed offspring, which may be then be maintained into postnatal life (21,22). Conversely, a recent study of DNA methylation in newborns found no difference in methylation between the offspring of mothers who never smoked and those who smoked early in pregnancy (23). It has also been shown that the effect of *in utero* exposure on newborn methylation is stronger when the mother smoked past 18 weeks than when she quit earlier in pregnancy (20). These findings warrant further investigation in an independent study.

Associations between own smoking and methylation at later time points have been found (15,24), with one study of former smokers showing that methylation in a key gene region associated with smoking (*AHRR*) approaches the levels of never smokers within the first few years of quitting, but never completely returns to normal levels (15). Two recent studies have also investigated prospective associations between maternal smoking in pregnancy and peripheral blood methylation in offspring when they were children (25) and adolescents (26). A high degree of similarity was found with smoking-associated DNA methylation in newborns (18), implying a lasting effect of maternal smoking in pregnancy on offspring DNA methylation profiles. However, a more comprehensive longitudinal assessment of intrauterine exposure and methylation levels in the same offspring assessed at multiple time points is required.

The relative roles of the intrauterine and postnatal environment in the persistence of DNA methylation changes associated with maternal smoking are yet to be determined. Previous studies have shown that associations between prenatal exposure to maternal cigarette smoking and offspring methylation during adolescence are not attenuated with adjustment for postnatal smoking of the parents or the offspring themselves (25,26). However, the method of adjusting for a potential mediator in standard regression models to estimate the direct effect of an exposure may produce spurious conclusions (27,28). Alternative methods are therefore required to test the hypothesis that maternal smoking in pregnancy is the ‘critical period’ for influencing offspring methylation profiles in childhood and adolescence (29). Finally, given that some of the signals found for prenatal smoke exposure have also been identified in a study of personal smoking of adolescents (30), any apparent persistent effect of maternal smoking on offspring methylation profile at later ages may be explained by smoking of the adolescents themselves.

Epigenetic markers, in contrast to germ-line genetic variation (31), are phenotypic and are therefore subject to the same potential problems of confounding which afflict observational epidemiology (32,33). Hence, there is a need to apply a range of tools for strengthening causal inference in epigenetic epidemiology (34,35). One such method for inferring a causal intrauterine effect involves the use of paternal exposures as negative controls for maternal exposures thought to have an intrauterine influence on offspring outcomes (34,36–40). Paternal smoking may show associations with offspring methylation similar to those of maternal smoking in pregnancy if the associations are confounded either by shared familial factors or by parental genotypes. However, if there is an intrauterine influence of maternal smoking, then only maternal exposure would be expected to show an independent association with the outcome.

We use the Accessible Resource for Integrated Epigenomic Studies (ARIES), a large collection of genome-wide DNA methylation data from multiple time points in mothers and offspring from the Avon Longitudinal Study of Parents and Children (ALSPAC) (41,42) to (1) replicate findings of a recently reported EWAS for maternal cotinine (18) by investigating associations between self-reported maternal smoking in pregnancy and offspring cord blood methylation using the Illumina Infinium^®^ HumanMethylation450 (HM450) BeadChip; (2) explore the dose-dependent effect of maternal smoking by investigating associations between the duration and intensity of maternal smoking and offspring cord blood methylation at key CpG sites; (3) examine the persistence of DNA methylation changes at key CpG sites by investigating longitudinal associations at multiple time points, from birth to 17 years; (4) investigate the relative roles of the intrauterine and postnatal environment in the persistence of DNA methylation modifications; (5) assess potential causality in associations between maternal smoking during pregnancy and offspring DNA methylation at multiple time points, using paternal smoking as a negative control.

## Results

### Baseline characteristics

Compared with offspring in the core ALSPAC sample who are not part of the ARIES project, those in ARIES were more likely to be singletons, had a higher birth weight on average, had a longer gestation and had mothers who were: older at time of delivery, more highly educated, from a higher social class, more likely to drink alcohol in pregnancy and less likely to smoke in pregnancy (Table 1).

**Table 1.** Baseline characteristics for individuals in ARIES compared with those in the ALSPAC core cohort who were not part of ARIES

	Individuals in ARIES (*N* = 1018)^a^	Individuals not in ARIES (*N* = 14 062)^a^	*P-*value for difference
Sex (% males)	48.8	51.7	0.056
Multiple births (% singletons)	99.6	97.1	<0.001
Birth weight (g)	3487.4 (488.1)	3377.1 (577.9)	<0.001
Gestational age (weeks)	39.6 (1.5)	39.3 (2.1)	<0.001
Maternal age (years)	30.0 (4.4)	28.3 (5.0)	<0.001
Maternal parity (% parous)	53.6	55.5	0.26
Maternal education (% university degree)	20.5	12.2	<0.001
Household social class (% non-manual)	88	79.8	<0.001
Maternal BMI (kg/m^2^)	22.8 (3.7)	22.9 (3.8)	0.18
Alcohol during pregnancy? (% yes)	66.7	63.1	0.024
Mother smoked during pregnancy? (% yes)	14.3	30.2	<0.001

^a^*N* varies according to completeness of data on baseline characteristics.

Of the 1018 mother–offspring pairs in the ARIES project, 916 offspring had cord blood methylation data, which successfully passed quality control (QC). Seven hundred and ninety had data on both sustained smoking in pregnancy and cord blood DNA methylation. Of these, 699 were classified as non-smokers and 91 were classified as sustained smokers during pregnancy. Compared with the non-smokers, sustained smokers were more likely to be younger at time of delivery, less well educated, from a lower social class, less likely to drink in pregnancy and more likely to have partners who also smoked in pregnancy (Table 2).

**Table 2.** Differences in potential confounding factors between individuals in ARIES whose mothers did not smoke in pregnancy compared with sustained smokers

	Non-smoker (*N* = 699)^a^	Sustained (*N* = 91)^a^	*P-*value for difference
Sex			
Male	49.4	47.3	0.71
Female	50.6	52.8	
Maternal age (years)	30.5 (4.1)	27.9 (5.4)	<0.001
Maternal age (categories)			
<25	7.6	30.8	<0.001
25–30	39.2	37.4	
>30	53.2	31.9	
Parity			
0	45.5	51.1	0.53
1	37.7	32.2	
2	13.2	11.1	
3+	3.6	5.6	
Maternal education			
CSE/vocational	13.9	33.0	<0.001
O-level	32.7	40.9	
A-level	30.4	18.2	
Degree	23.1	8.0	
Social class			
I	22.9	4.9	<0.001
II	44.0	39.0	
III (NM)	24.4	20.7	
III (M)	5.3	22.0	
IV or V	3.5	13.4	
Maternal BMI	22.8 (3.7)	23.0 (3.6)	0.63
Maternal BMI (categories)			
<18.5	3.3	3.5	0.59
18.5–25	79.1	76.5	
25–30	13.1	14.1	
30+	4.6	5.9	
Maternal weight	61.6 (10.4)	61.5 (10.5)	0.95
Alcohol			
Non-drinker	34.2	40.9	0.040
Drank before 18 weeks of gestation	15.6	5.7	
Still drinking at 18 weeks of gestation	50.2	53.4	
Paternal smoking			
Non-smoker	78.6	20.9	<0.001
Smoker	21.4	79.1	

^a^*N* varies according to completeness of data on baseline characteristics.

### EWAS for maternal smoking in pregnancy and cord blood methylation

**Figure 1.**
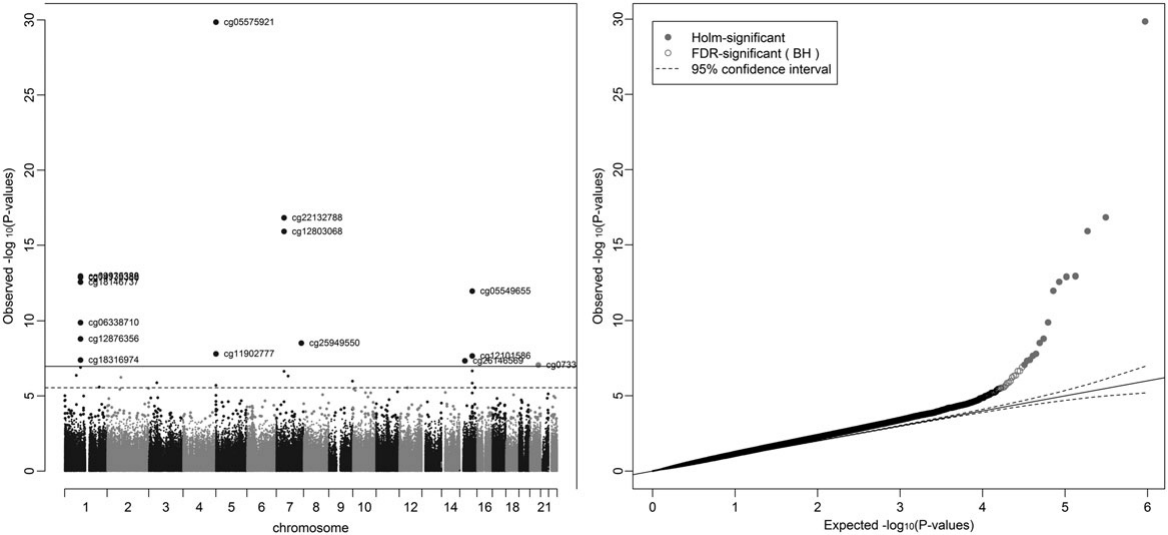
Manhattan and QQ plot for epigenome-wide association study of sustained smoking in pregnancy on cord blood DNA methylation. *Results are from the analysis adjusted for batch only (*N* = 790).

In an unadjusted analysis of the associations between maternal smoking in pregnancy and cord blood epigenome-wide methylation levels, 15 CpG sites fell below the Bonferroni threshold for significance of 1.07 × 10^−7^ and 28 CpG sites fell below the false discovery rate (FDR) cut-off of 0.05 (Fig. 1 and Table 3). Of the CpG sites falling below the Bonferroni threshold, these were located in seven gene regions and most have been previously identified in EWAS for maternal smoking, with the top hit in *AHRR* (cg05575921) being consistently replicated (18,19). The effects of smoking on methylation levels were directionally consistent with previous studies (23,24) for all of these sites, with hypomethylation of sites at *AHRR*, *GFI1* and *CNTNAP2* and hypermethylation of *MYO1G* and *CYP1A1* in the offspring of smokers compared with non-smokers. Of the CpG sites which fell below the FDR but not the Bonferroni threshold, five of these CpG sites were also located in the *AHRR*, *GFI1*, *CYP1A1* and *MYO1G* gene regions. Other gene regions harbouring CpG sites associated with maternal smoking at Bonferroni significance were *KLF13* and *ATP9A* and at FDR significance were *GNG12*, *ENSG00000225718*, *CTNNA2*, *NOTCH1*, *ALS2CL*, *CHI3L1*, *ZNF710* and *SPATS2*. Sites at *ATP9A*, *GNG12* and *ENSG00000225718* have previously identified in other EWAS for maternal smoking (18,19), but the other sites appear to be novel.

**Table 3.** Differential methylation in cord blood DNA for the offspring of mothers with sustained smoking in pregnancy compared with non-smokers

CpG site	Chromosome	Gene region	Position^c^	Unadjusted model (*N* = 790^†^)^a^				Adjusted model (*N* = 744^‡^)^b^			
				Effect size	Standard error	*P-*value	FDR	Effect size	Standard error	*P-*value	FDR
cg05575921	5	*AHRR*	373 378	−0.081	0.006	1.41 × 10^−30^	6.59 × 10^−25^	−0.075	0.007	7.64 × 10^−18^	3.56 × 10^−12^
cg22132788	7	*MYO1G*	45 002 486	0.062	0.010	1.49 × 10^−17^	3.47 × 10^−12^	0.054	0.012	1.72 × 10^−10^	3.03 × 10^−5^
cg12803068	7	*MYO1G*	45 002 919	0.152	0.021	1.22 × 10^−16^	1.90 × 10^−11^	0.126	0.025	4.47 × 10^−9^	3.47 × 10^−4^
cg09935388	1	*GFI1*	92 947 588	−0.167	0.023	1.14 × 10^−13^	1.19 × 10^−8^	−0.174	0.028	1.95 × 10^−10^	3.03 × 10^−5^
cg14179389	1	*GFI1*	92 947 961	−0.078	0.012	1.28 × 10^−13^	1.19 × 10^−8^	−0.070	0.015	2.49 × 10^−8^	1.45 × 10^−3^
cg18146737	1	*GFI1*	92 946 700	−0.144	0.019	2.67 × 10^−13^	2.07 × 10^−8^	−0.141	0.024	2.10 × 10^−9^	1.96 × 10^−4^
cg05549655	15	*CYP1A1*	75 019 143	0.015	0.002	1.05 × 10^−12^	6.99 × 10^−8^	0.016	0.002	4.73 × 10^−10^	5.51 × 10^−5^
cg06338710	1	*GFI1*	92 946 187	−0.214	0.032	1.32 × 10^−10^	7.69 × 10^−6^	−0.200	0.039	5.93 × 10^−7^	0.02
cg12876356	1	*GFI1*	92 946 825	−0.179	0.029	1.60 × 10^−9^	8.27 × 10^−5^	−0.206	0.035	1.36 × 10^−8^	9.09 × 10^−4^
cg25949550	7	*CNTNAP2*	145 814 306	−0.006	0.001	3.06 × 10^−9^	1.43 × 10^−4^	−0.006	0.001	8.08 × 10^−6^	0.09
cg11902777	5	*AHRR*	3 68 843	−0.005	0.001	1.59 × 10^−8^	6.74 × 10^−4^	−0.004	0.001	5.62 × 10^−5^	0.17
cg12101586	15	*CYP1A1*	75 019 203	0.060	0.010	2.18 × 10^−8^	8.49 × 10^−4^	0.070	0.013	5.71 × 10^−8^	2.96 × 10^−3^
cg18316974^^d^^	1	*GFI1*	92 947 035	−0.083	0.019	4.05 × 10^−8^	1.45 × 10^−3^	−0.086	0.023	4.96 × 10^−6^	0.08
cg26146569	15	*KLF13*	31 637 592	−0.072	0.013	4.61 × 10^−8^	1.54 × 10^−3^	−0.083	0.016	2.97 × 10^−7^	0.01
cg07339236^^d^^	20	*ATP9A*	50 312 490	−0.016	0.004	8.71 × 10^−8^	2.71 × 10^−3^	−0.014	0.006	3.60 × 10^−4^	0.24
cg09662411	1	*GFI1*	92 946 132	−0.127	0.024	1.26 × 10^−7^	3.66 × 10^−3^	−0.147	0.029	6.60 × 10^−7^	0.02
cg18092474	15	*CYP1A1*	75 019 302	0.077	0.016	2.17 × 10^−7^	5.94 × 10^−3^	0.057	0.019	5.12 × 10^−4^	0.24
cg04180046	7	*MYO1G*	45 002 736	0.050	0.011	2.29 × 10^−7^	5.94 × 10^−3^	0.043	0.012	4.23 × 10^−5^	0.16
cg25189904	1	*GNG12*	68 299 493	−0.055	0.011	4.32 × 10^−7^	0.01	−0.052	0.013	1.20 × 10^−4^	0.20
cg04598670	7	*ENSG00000225718*	68 697 651	−0.074	0.015	4.80 × 10^−7^	0.01	−0.074	0.018	4.35 × 10^−5^	0.16
cg27629977	2	*CTNNA2*	80 531 633	0.009	0.002	5.82 × 10^−7^	0.01	0.012	0.003	4.07 × 10^−7^	0.01
cg10835306	9	*NOTCH1*	139 396 760	−0.079	0.016	1.04 × 10^−6^	0.02	−0.061	0.019	2.34 × 10^−3^	0.30
cg00483459	3	*ALS2CL*	46 735 782	−0.049	0.010	1.32 × 10^−6^	0.03	−0.056	0.011	2.19 × 10^−6^	0.05
cg22549041^^d^^	15	*CYP1A1*	75 019 251	0.069	0.014	1.42 × 10^−6^	0.03	0.089	0.017	2.60 × 10^−7^	0.01
cg22937882^^d^^	5	*AHRR*	4 05 774	0.036	0.008	1.98 × 10^−6^	0.04	0.036	0.011	3.13 × 10^−4^	0.24
cg11196333	1	*CHI3L1*	203 154 370	−0.060	0.013	2.58 × 10^−6^	0.05	−0.066	0.015	1.06 × 10^−5^	0.11
cg00624799^^d^^	15	*ZNF710*	90 605 618	−0.029	0.006	2.79 × 10^−6^	0.05	−0.037	0.007	4.19 × 10^−7^	0.01
cg00560284	12	*SPATS2*	49 783 222	−0.016	0.003	2.84 × 10^−6^	0.05	−0.013	0.004	1.79 × 10^−3^	0.29

Effect size = difference in methylation level (beta) between offspring of sustained smokers and non-smokers.

^a^Adjusted for batch only: ^†^*N* = 91 sustained smokers; *N* = 699 non-smokers (defined as those who did not smoke pre-pregnancy or in pregnancy).

^b^Adjusted for maternal age, maternal education, household social class, paternal smoking, maternal alcohol in pregnancy and batch: ^‡^*N* = 76 sustained smokers; *N* = 668 non-smokers (defined as those who did not smoke pre-pregnancy or in pregnancy).

^c^Chromosomal position based on NCBI human reference genome assembly Build 37.3.

^d^Naeem flagged CpG site (43).

The sample size was reduced to 744 participants once all covariates were included in the adjusted model. Results were slightly attenuated in the model adjusting for a number of potential confounding factors and 12 probes no longer reached the FDR cut-off for epigenome-wide significance (Table 3). This reduction in the number of CpG sites reaching epigenome-wide significance with adjustment for confounders is likely due to a loss of power with a reduced sample size, because the magnitude and direction of methylation difference at all the sites were similar.

We next investigated whether any of the CpG sites that reached epigenome-wide significance in our main analysis were identified as being either single nucleotide polymorphism (SNP)-confounded or cross-hybridizing based on a comprehensive assessment reported by Naeem *et al.* (43). Five CpG sites identified in the original analysis were flagged by this study as sites to exclude as SNPs are known to overlap the probe region (Table 3).

Evidence for a difference in four of the six estimated cell proportions was found between non-smokers and sustained smokers (Supplementary Material, Table S1). To establish the effect of correcting for cell type, we added the predicted cell-type components as covariates in the main model. Results were largely unaltered with this adjustment (Supplementary Material, Table S2).

We also explored whether there were any sex-specific associations by stratifying the analysis based on sex of the offspring (Supplementary Material, Table S3). This analysis involved 388 boys and 402 girls. In boys, three CpG sites reached the FDR threshold for epigenome-wide significance, located in *AHRR*, *MYO1G* and *CYP1A1*. In girls, three CpG sites reached the FDR threshold for epigenome-wide significance, located in *AHRR*, *MYO1G* and *GFI1*. These same sites were among the top hits in the combined analysis. There was some evidence for an interaction by sex at *AHRR* (cg05575921), where the methylation change associated with sustained smoking was larger in girls than in boys and at *CYP1A1* (cg05549655) where the methylation change was larger in boys than in girls. However, there was limited evidence for a difference in effect size between boys and girls at the other CpG sites in these same gene regions, providing no strong evidence for sex-specific associations.

Given that most of the CpG sites falling below the Bonferroni threshold were located within common genomic regions, we used coMET (44), a web-based plotting tool, to visualize the genomic regions of interest from our EWAS (Supplementary Material, Figs S1–S7). There was some evidence for localized clustering around the top CpG site (that with the smallest *P*-value in the EWAS) in *AHRR*, *MYO1G*, *GFI1* and *CYP1A1*, although there was little evidence for strong co-methylation within the gene regions indicating independence in methylation levels at each CpG site. However, we decided to only take forward the CpG site with the smaller *P*-value in each gene region to focus our downstream analyses.

### Dose-dependence of cord blood methylation on maternal smoking

**Figure 2.**
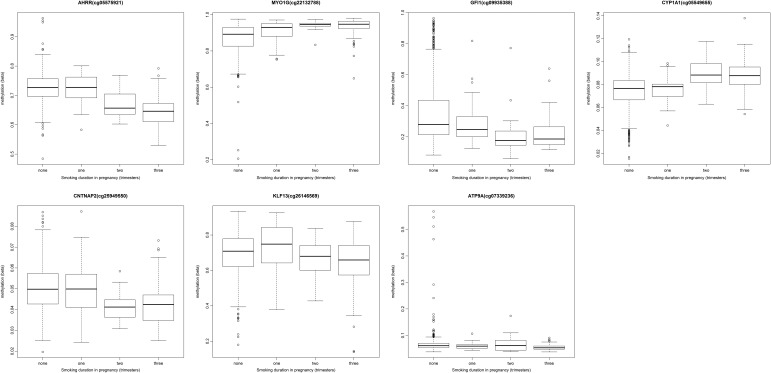
Methylation level (beta) at key CpG sites associated with sustained smoking in pregnancy by the duration of smoking (number of trimesters).

**Figure 3.**
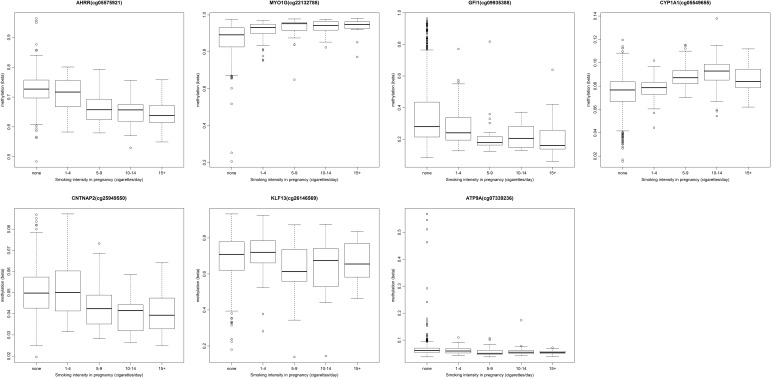
Methylation level (beta) at key CpG sites associated with sustained smoking in pregnancy by the intensity of smoking (number of cigarettes per day).

To investigate dose-dependent effects of maternal smoking on cord blood methylation in the offspring, we ran an exploratory analysis for the top CpG sites in each of the seven gene regions identified in the main combined analysis: *AHRR* (cg05575921), *MYO1G* (cg22132788), *GFI1* (cg09935388), *CYP1A1* (cg05549655), *CNTNAP2* (cg25949550), *KLF13* (cg26146569) and *ATP9A* (cg07339236). We found that cord blood methylation differences between the offspring of mothers who smoked in pregnancy compared with that of non-smokers were more extreme with both increased duration (number of trimesters; Fig. 2) and intensity (average number of cigarettes per day) of smoking in pregnancy (Fig. 3), though this trend was more pronounced at some sites than others, e.g. *AHRR* (cg05575921) (*P* = 2.7 × 10^−42^) versus *ATP9A* (cg07339236) (*P* = 9.9 × 10^−3^) for the duration of smoking in pregnancy.

### Longitudinal analysis of maternal smoking in pregnancy and offspring methylation

**Figure 4.**
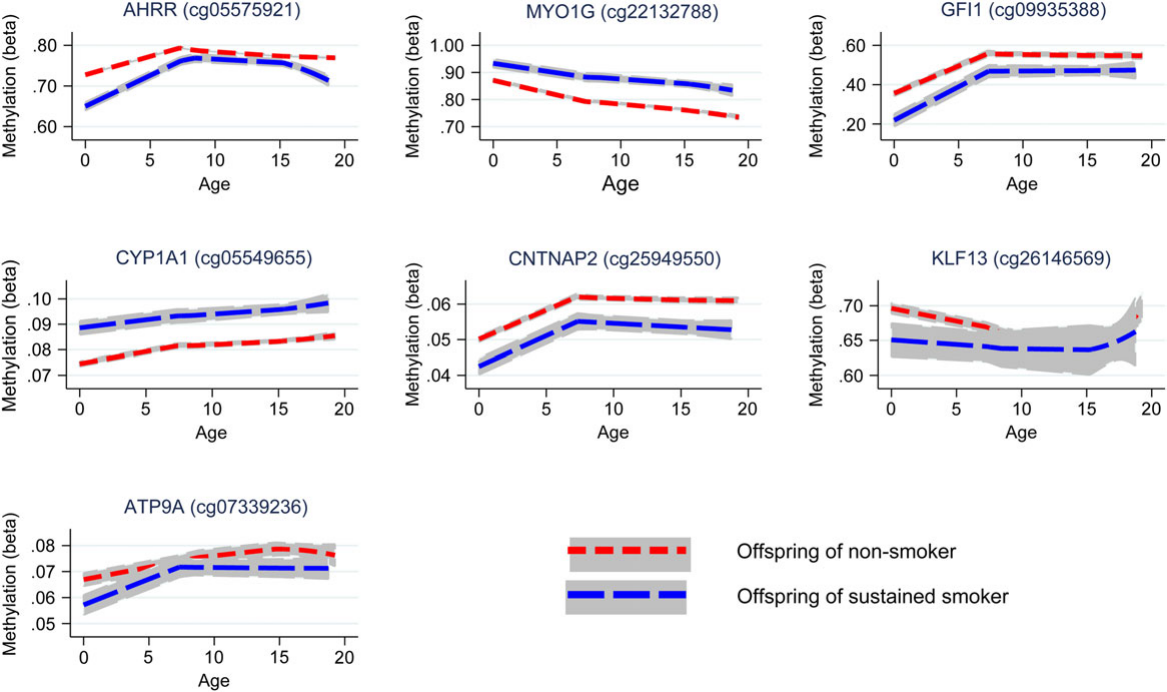
Longitudinal trajectories of methylation at key CpG sites in the offspring of non-smokers and sustained smokers during pregnancy from birth to age 17.

Longitudinal analyses were performed to investigate whether the effect of smoking on offspring methylation at birth was transient or persisted into later life. Methylation data were available for offspring in ARIES at age 7 [mean age when blood samples were taken 7.5 (SD 0.1)] and at age 17 [mean age 17.1 (SD 1.0)]. We investigated changes in methylation levels for the CpG sites that were found to be associated with maternal smoking in cord blood using multilevel modelling (Fig. 4 and Supplementary Material, Table S4). For the seven CpG sites, there were changes in methylation found during childhood, while the magnitude of change was quite small during adolescence. At *CYP1A1* (cg05549655) and *CNTNAP2* (cg25949550), while there was some evidence for change in methylation among the offspring of the smokers and non-smokers over time, the difference in methylation between groups persisted. Evidence for differing rates of change in methylation level between the offspring of smokers and non-smokers was found at *AHRR* (cg05575921), *MYO1G* (cg22132788), *GFI1* (cg09935388) and *KLF13* (cg26146569) between birth and age 7 (*P*-value for difference in methylation change 0.01–1 × 10^−16^).

At *MYO1G* (cg22132788), methylation level for the offspring of smokers deviated more from the level of the offspring of non-smokers over time, whereas at *GFI1* (cg09935388) and *KLF13* (cg26146569) there was some recovery of methylation towards the level of those not exposed to prenatal maternal smoke. At *AHRR* (cg05575921) during childhood, methylation increased at a faster rate in the offspring of smokers with evidence for a ‘catchup’ in methylation among the offspring of smokers [a 2.04% (95% CI 1.72, 2.36%) average yearly increase in methylation for the offspring of sustained smokers compared with a 1.28% (95% CI 0.97, 1.59%) increase in methylation for the offspring of non-smokers, between birth and age 7]. However, during adolescence, levels of *AHRR* (cg05575921) methylation decreased among both the smoker and non-smoker offspring, with methylation in the smoker offspring decreasing at a faster rate [a 0.33% (95% CI 0.26, 0.40%) average yearly decrease in methylation for the offspring of sustained smokers compared with a 0.17% (95% CI 0.12, 0.22%) decrease in methylation for the offspring of non-smokers, between age 7 and 17], leading again to a difference in methylation levels. A similar trend was found for *ATP9A* (cg07339236), although this was not as robust.

For the CpG sites which showed a persistent difference in methylation between the offspring of smokers and non-smokers [*MYO1G* (cg22132788), *CYP1A1* (cg05549655) and *CNTNAP2* (cg25949550); Fig. 4], we sought to determine whether the associations with maternal smoking were explained by a direct ‘critical period’ effect of smoking in pregnancy or via an indirect pathway involving postnatal smoke exposure. Identifying these underlying mechanisms of association is hampered by the high correlation (0.87) between sustained smoking in pregnancy and maternal smoking at 8 weeks postnatally. To disentangle the effect of smoking in pregnancy on offspring methylation versus smoking postnatally, we implemented a structured approach to model the effects of the binary maternal smoking exposure at three time points (in pregnancy and postnatally at 8 weeks and 61 months) on offspring methylation at age 7 (Table 4). The hypothesis for an *in utero* critical period is supported by data at all three CpG sites, with this model not being substantially different from the saturated model (*P* ≥ 0.06). However, a model for effect modification of the intrauterine exposure was also supported by the data (*P* ≥ 0.06) and for *CYP1A1* and *CNTNAP2* a critical period at 8 weeks postnatally could not be ruled out (*P* ≥ 0.18). As the *in utero* critical period model is nested within the effect modification model, we further performed a direct ANOVA test to investigate whether effect modification provided a better fit of the data than the *in utero* critical period model. This was found to be the case for *CYP1A1* (*P* = 0.009), but not for *MYO1G* or *CNTNAP2* (*P* ≥ 0.22), where the *in utero* critical period was found to be the best model (Table 4).

**Table 4.** An exploration of critical period and other lifecourse effects that may underlie the persistence of associations between maternal smoke exposure in pregnancy and offspring methylation at age 7

Lifecourse hypothesis	MYO1G (cg22132788)		CYP1A1 (cg05549655)		CNTNAP2 (cg25949550)	
	*F*-statistic	*P*-value	*F*-statistic	*P*-value	*F*-statistic	*P*-value
Critical period in pregnancy	0.68	0.66	1.62	0.14	2.05	0.06
Critical period at 8 weeks postnatally	3.74	0.001	1.48	0.18	1.00	0.42
Critical period at 61 months postnatally	4.72	9.74 × 10^−5^	5.07	4.02 × 10^−5^	3.35	0.003
Accumulation of risk over time	3.89	7.77 × 10^−4^	5.83	5.77 × 10^−6^	4.19	3.68 × 10^−4^
Effect modification of critical period in pregnancy by postnatal exposure	0.82	0.51	0.08	0.98	2.32	0.06
Critical period in pregnancy nested within effect modification model^a^	0.40	0.67	4.8	0.009	1.50	0.22

Results of ANOVA test against a saturated model; a smaller *F-*statistic (and larger *P*-value) provides evidence of a better fit of data to the hypothesized model.

^a^Results of ANOVA test against effect modification model.

For the CpG sites which showed evidence of methylation difference between the offspring of smokers and non-smokers at age 17 [*AHRR* (cg05575921), *MYO1G* (cg22132788), *CYP1A1* (cg05549655) and *CNTNAP2* (cg25949550); Fig. 4], we followed up the association between methylation at these sites and own smoking among the adolescents (Supplementary Material, Table S4). The correlation between sustained smoking in pregnancy and own smoking (of the adolescent) was found to be 0.16. For the majority of CpG sites for which the association with smoking in pregnancy was evident at age 17, there was directional consistency of the association between own smoking and methylation at this time point*.* However, the magnitude of the association with own smoking was smaller than for the sustained maternal prenatal smoking analysis at all CpG sites, with the exception of *AHRR* (cg05575921), where the effect size for sustained smoking in pregnancy was −3.6% (95% CI −4.6, −2.6%) compared with −3.4% (95% CI −4.4, 2.4%) for the own smoking analysis, suggesting that not all of the association between maternal smoking in pregnancy and age 17 methylation can be explained through the mediating role of the adolescent's own smoking.

We next investigated the associations between sustained smoking in pregnancy and methylation at age 17, excluding those offspring who reported smoking themselves (Supplementary Material, Table S5). The magnitude and direction of association of the CpG sites were comparable to those in the full analysis, providing more evidence that own smoking is not fully mediating the observed association between maternal smoking in pregnancy and offspring methylation at age 17. In addition, this was with the notable exception of CpG sites at *AHRR*, where the effect size was halved [from −3.6% (95% CI −4.6, −2.6%) to −1.8% (95% CI 0.0, −3.6%)] when adolescents who reported smoking were excluded from the analysis. This provides some indication that personal smoking by adolescents and its correlation with maternal smoking could be driving the apparent persistent methylation difference in this gene region at age 17.

### Assessing causality of intrauterine associations using paternal smoking as a negative control

**Figure 5.**
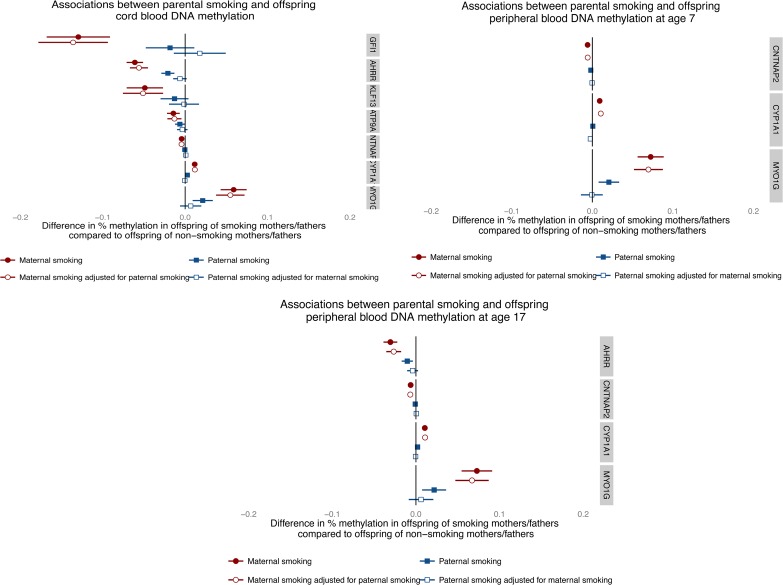
Parental comparisons of associations between any versus no smoking in pregnancy on offspring methylation at CpG sites most associated with sustained maternal smoking in pregnancy at all three time points.

Finally, parental comparisons of associations between smoking during pregnancy and methylation levels at the top CpG sites showed consistently larger effect estimates for prenatal maternal smoking than for paternal smoking at all three time points (Fig. 5). In addition, adjusting for paternal smoking in maternal associations made little difference to affect estimates while adjusting for maternal smoking attenuated all paternal associations. For example, in the analysis of methylation in cord blood, any smoking by mothers during pregnancy was associated with a 6.1% (95% CI −7.1, −5.1%) reduction in cord blood methylation at cg05575921 (*AHRR*), which was not substantially attenuated with adjustment for partner's smoking (−5.6%; 95% CI −6.7, −4.5%). Smoking by partners during pregnancy was associated with a 2.1 (−2.8, −1.3%) reduction in cord blood methylation at cg05575921 (*AHRR*), which was fully attenuated with adjustment for maternal smoking (−0.01%; 95% CI −0.01, 0.00%).

## Discussion

In a large longitudinal birth cohort with genome-wide methylation measured at three different time points in the offspring, we first identified 15 CpG sites that were differentially methylated in cord blood at birth. These sites are located in seven gene regions, six of which have been previously identified in other EWAS for exposure to maternal smoking *in utero* (18,19). The top hit in this analysis was located within the *AHRR* [aryl hydrocarbon receptor (AHR) repressor] gene. CpG sites located in this gene region have previously been shown to be differentially methylated in smokers in several studies (13–18,30). In particular, the top hit in this analysis (cg05575921, *P* = 1.41 × 10^−30^) was identified in previous studies including an epigenome-wide association study for maternal smoking and both cord and neonatal blood DNA methylation (18).

At this site, an 8.1% (95% CI 6.9, 9.3%) reduction in cord blood methylation with sustained prenatal smoking exposure was identified, which is in line with the median methylation difference of medium and high cotinine versus no exposure in a previous EWAS (18), which was 5.4 and 9.9%, respectively. These associations were largely robust to adjustment for a number of genetic, environmental and cell-type specific confounding factors, supporting a causal effect of maternal smoking during pregnancy on offspring methylation at birth. However, for two of the CpG sites followed up in downstream analysis, *CNTNAP2* (cg25949550) and *ATP9A* (cg07339236), the FDR *P*-values were 0.09 and 0.24, respectively, in the adjusted model, but effect estimates were largely unchanged between the adjusted and unadjusted model. In addition, *ATP9A* (cg07339236) was flagged up in Table 3 as a low-quality probe based on a comprehensive assessment reported by Naeem *et al.* (43).

A regional analysis of EWAS hits provided some evidence for localized clustering around the top CpG site (that with the smallest *P*-value in the EWAS) in *AHRR*, *MYO1G*, *GFI1* and *CYP1A1*, although there was little evidence for strong co-methylation within the gene regions indicating independence of methylation levels at each CpG site, supporting our use of single site analysis in the EWAS.

We assessed the biological gradient of smoke exposure in pregnancy and identified a dose-dependent response of methylation with both increased intensity and duration of smoking. In this analysis, we found that methylation in the offspring of mothers who smoked only in one trimester, namely the first, was largely comparable to that of unexposed offspring. These findings are in line with previous studies, which showed no difference in mean methylation at *AHRR* between mothers who never smoked and those who smoked early in their pregnancy (20,23), suggesting that sustained exposure to maternal smoking *in utero* is required to induce changes in methylation which are detectable in cord blood. In contrast to the view that early pregnancy represents a critical window for environmentally induced epigenetic change, epigenetic reprogramming appears to occur throughout prenatal development and postnatally (45) and these findings imply a cumulative effect of smoke exposure throughout pregnancy on offspring methylation at birth.

Nonetheless, knowledge of smoking intensity in the first trimester is important as a predictive marker of smoking later in pregnancy and hence of epigenetic change in the offspring. This has been confirmed in an analysis of 374 ARIES mothers–child pairs for whom urinary cotinine was collected in the first trimester of pregnancy. An EWAS of maternal cotinine levels and cord blood methylation in this subsample was able to identify a signal at *AHRR* (cg05575921), which surpassed the Bonferroni threshold (*P*-value = 3.31 × 10^−8^; Supplementary Material, Fig. S8).

Whether pregnancy represents a critical period for determining offspring methylation patterns at later time points in childhood in response to maternal smoking was also investigated. A longitudinal assessment of methylation marks associated with maternal smoke exposure in pregnancy found that whereas some CpG sites showed recovery of methylation to the level of offspring not exposed (*GFI1*, *KLF13* and *ATP9A*), other sites showed persistently perturbed patterns (*AHRR*, *MYO1G*, *CYP1A1* and *CNTNAP2*).

This prospective study design coupled with serial sampling at multiple time points provides powerful evidence of the persistence of DNA methylation changes induced *in utero*. In addition, longitudinal modelling of the effects of exposure ‘windows’ provides evidence that prenatal exposure to smoking has persistent effects on later offspring DNA methylation, which outweighs the postnatal influence of maternal smoking or own smoking in adolescence at some CpG sites. Effect modification of prenatal exposure at later time points was also evident, indicating that postnatal exposure might have some further impact on the persistence of the methylation marks in those exposed to smoking *in utero*. However, there was no strong evidence that the effect modification model was more consistent with the observed data than the *in utero* critical period model at most sites. These observations are consistent with and significantly extend previous analyses of long-term smoking-induced perturbations of DNA methylation (15,23,24,26). One exception to this is the finding that own smoking by the offspring at age 17 is strongly associated with *AHRR* methylation at this time point which might therefore be underlying the apparently persistent effect of maternal smoking in pregnancy at this gene region, as shown in Figure 4. This is perhaps because this site is most sensitive to smoking exposure and would therefore detect adolescent own smoking most readily.

In addition, the use of paternal smoking as a negative control demonstrates the biological effect of this *in utero* exposure at all time points considered. While methylation differences identified between maternal and paternal smoking at later time points might be attributed to the differential influences of these exposures postnatally, the similar trends identified in cord blood when no influence of the postnatal environment had been present provide further support for the causal effect of maternal smoking in pregnancy on offspring methylation. In addition, the low levels of tobacco exposure from partner smoking in non-smoking pregnant women in this cohort suggest that the use of partner's smoking as a negative control for investigating intrauterine effects is valid (40). Mendelian randomization is another technique that may be used to bolster causal inference in this context (35,46,47). A SNP, rs1051730, located in the *CHRNA5-CHRNA3-CHRNB4* nicotinic acetylcholine receptor gene cluster (chromosome 15q25), is robustly associated with smoking heaviness (48) and has also been associated with a reduced ability of women to quit smoking in pregnancy (49). If an association was observed between maternal rs1051730 and offspring DNA methylation of mothers who smoked during pregnancy, this would provide further evidence of an intrauterine effect. However, we were underpowered to investigate this formally within our sample of sustained smokers.

Strengths of our study include the application of the Illumina Infinium^®^ HM450 BeadChip technology to assess genome-wide methylation profiles at multiple time points from birth until late adolescence in a large, longitudinal cohort study. The wealth of phenotypic data in ALSPAC has aided the thorough assessment of potential confounding factors, a detailed analysis of the dose-dependence of methylation to smoke exposure *in utero* and an investigation into the relative roles of intrauterine and postnatal smoking using questionnaire data on smoking habits taken from multiple time points in both parents and offspring. Longitudinal modelling (50,51) and robust statistical methods (34,39,52) have also been used to strengthen causal inference.

Limitations of the analysis include differential follow-up of smokers compared with non-smokers, where only 14.3% of mothers in the ARIES sample (selected based on blood sample availability up to 17 years postnatally) smoked in pregnancy compared with 30.2% in the wider cohort. Technical limitations relate to the HM450 BeadChip in that it covers only 1.7% of CpG sites across the genome. A more comprehensive appraisal may elicit additional relationships between the exposure in question and DNA methylation, or indeed locus-specific paternal effects which were not evident here (53–55). In addition, the use of questionnaires to obtain data on parental smoking may result in under-reporting of smoking behaviour. However, to minimize the influence of maternal under-reporting, we excluded from the analyses individuals who had reported smoking before but not during pregnancy. In addition, a strong correlation between self-reported questionnaire data on smoking behaviour and plasma cotinine levels has been found (18,40) and maternal cotinine was found to be highly correlated with the sustained smoking variable (*r* = 0.76) in the subsample of ARIES participants with first trimester maternal cotinine data available (*N* = 323).

This analysis was limited to blood samples with mixed cell composition. Although no differences were found in the analysis with estimated cell-type correction, as has been shown previously (18,19), it is unclear how effective the method used to correct for cell-type proportions is in these samples since the reference data sets are available only for adult peripheral blood (56). In addition, some of the DNA samples included in this analysis came from buffy coats rather than whole blood and there is no reference cell-type correction available for buffy coat DNA. It should also be emphasized that the associations identified may be specific to blood as an analysis of buccal epithelium and placenta did not identify the same smoking-associated methylation differences in these tissues (23). This limitation of tissue specificity, as well as the lack of expression data currently available on these samples, limits the assessment of functional consequences of these methylation changes.

Given evidence for causal associations between maternal smoking in pregnancy and methylation changes in the offspring, it is important to consider whether these induced changes are also associated with the adverse perinatal and offspring outcomes associated with exposure to smoking *in utero*. Further work is required which may link smoking-responsive DNA methylation variation to health and development (57–59). In addition, whether DNA methylation is a true mediating mechanism of these associations or simply an exposure indicator may be explored by extending causal inference (35,47,60).

Transient environmental exposures during critical windows of development are known to affect the establishment of epigenetic marks, which are evident at birth (57) and may persist until later life (58,59). Findings from this study highlight the sensitivity of the methylome to maternal smoking during fetal development and the long-term impact of such an exposure. Results strengthen causal inference in this area and the finding that sustained smoking appears to be necessary to induce methylation changes has profound implications for antenatal care and the long-term effects on offspring health, directing potential intervention strategies at cessation of smoking early in pregnancy, such as at the first antenatal appointment.

These findings could also have very useful applications in epidemiological studies. Given the magnitude and persistence of methylation change in relation to maternal exposure *in utero*, it is important to consider the potential confounding effect of maternal smoking in future EWAS studies attempting to identify sites associated with own smoking. The persistence of some (but not all) methylation marks at later time points presents the opportunity to use methylation signatures as an archive of historical exposure, particularly if methylation patterns can be robustly modelled over time and previous exposure inferred. A simplistic example would be to define prenatal smoking exposure, using DNA methylation signatures in children of women where no smoking history had been collected. In addition, the contrast between stable and reversible sites may be useful in discriminating between *in utero* exposure and later life exposure.

## Materials and Methods

### Study design

We examined offspring DNA methylation in relation to self-reported maternal smoking during pregnancy in a subset of participants from ALSPAC using methylation data from the Illumina Infinium^®^ HumanMethylation450 BeadChip assay (Illumina, Inc., CA, USA) (61).

### Cohort and selection of participants

ALSPAC is a large, prospective cohort study based in the South West of England. A total of 14 541 pregnant women resident in Avon, UK, with expected dates of delivery 1 April 1991 to 31 December 1992 were recruited and detailed information has been collected on these women and their offspring at regular intervals (41,42). The study website contains details of all the data that are available through a fully searchable data dictionary (http://www.bris.ac.uk/alspac/researchers/data-access/data-dictionary/).

As part of the ARIES (http://www.ariesepigenomics.org.uk/) project, the Infinium HM450 BeadChip has been used to generate epigenetic data on 1018 mother–offspring pairs in the ALSPAC cohort. The ARIES participants were selected based on availability of DNA samples at two time points for the mother (antenatal and at follow-up when the offspring was in adolescence) and at three time points for the offspring [neonatal, childhood (age 7) and adolescence (age 17)].

Written informed consent has been obtained for all ALSPAC participants. Ethical approval for the study was obtained from the ALSPAC Ethics and Law Committee and the Local Research Ethics Committees.

### Laboratory methods, quality control and pre-processing

Cord blood and peripheral blood samples (whole blood, buffy coats or blood spots) were collected according to standard procedures. The DNA methylation wet laboratory and pre-processing analyses were performed at the University of Bristol as part of the ARIES project. Following extraction, DNA was bisulphite-converted using the Zymo EZ DNA MethylationTM kit (Zymo, Irvine, CA, USA). Following conversion, genome-wide methylation status of over 485 000 CpG sites was measured using the Infinium HM450 BeadChip according to the standard protocol. The arrays were scanned using an Illumina iScan and initial quality review was assessed using GenomeStudio (version 2011.1).

The Infinium HM450 BeadChip assay detects the proportion of molecules methylated at each CpG site on the array. For the samples, the methylation level at each CpG site was calculated as a beta value (*β*), which is the ratio of the methylated probe intensity and the overall intensity and ranges from 0 (no cytosine methylation) to 1 (complete cytosine methylation) (62,63). Methylation data were pre-processed using in R (version 3.0.1), with background correction and subset quantile normalization performed using the pipeline described by Touleimat and Tost (64).

Samples from all time points in ARIES were distributed across slides using a semi-random approach (sampling criteria were in place to ensure that all time points were represented on each array) to minimize the possibility of confounding by batch effects. In addition, during the data generation process a wide range of batch variables were recorded in a purpose-built laboratory information management system (LIMS). The main batch variable was found to be the bisulphite conversion (BCD) plate number. Samples were converted in batches of 48 samples and each batch identified by a plate number.

The LIMS also reported QC metrics from the standard control probes on the 450K BeadChip for each sample. Samples failing QC (average probe *P*-value of ≥0.01) were repeated and if unsuccessful excluded from further analysis. As an additional QC step genotype probes were compared with SNP-chip data from the same individual to identify and remove any sample mismatches. For individuals with no genome-wide SNP data, samples were flagged if there was a sex-mismatch based on X-chromosome methylation.

In addition to these QC steps, probes that contained <95% of signals detectable above background signal (detection *P*-value of <0.01; *N* = 7938) were excluded from analysis. After excluding these probes, as well as control probes and probes on sex chromosomes, a total of 466 432 CpG sites were included in the main analysis for cord blood methylation. At age 7, 471 347 CpG sites were included and at age 17, 469 902 CpG sites were included in the main analysis, following the same exclusion criteria.

### *In utero* exposure variables

Information on mothers' smoking status during pregnancy was obtained in questionnaires administered at 18 and 32 weeks of gestation. Information was obtained about whether the mother smoked in each trimester of pregnancy and the number of cigarettes smoked on average per day. From these data, a dichotomous variable for sustained maternal smoking during pregnancy was derived. A mother was classified as a sustained smoker if she smoked in all three trimesters, smoked in the first and third trimester but not the second or smoked in the second and third trimesters but not the first. The reference group consisted of mothers who had reported not smoking in all three trimesters or before pregnancy. We excluded all individuals who smoked in one trimester only (i.e. not sustained) or who had missing information of smoking for two or more trimesters. Of those with missing information on one trimester, women were classified as a sustained smoker if they said they smoked in the other two trimesters.

For investigating the dose-dependent effects of maternal smoking in pregnancy on DNA methylation in cord blood, a variable was derived for the duration of smoking in pregnancy (0, 1, 2 or all three trimesters) as well as the intensity of smoking in pregnancy (0, 1–4, 5–9, 10–14 and 15+ cigarettes/day).

Data on cotinine levels were available for a small subset of the ARIES mothers (*n* = 374). Cotinine levels (ng/ml) were assessed from a single urine sample taken during the first trimester of pregnancy. For most mothers, the samples were collected as part of routine clinical care but some samples were obtained specifically for ALSPAC. Urine samples were stored at −20°C and allowed to thaw at room temperature before use. Cotinine was measured using the Cozart Cotinine Enzyme Immunoassay (Concateno UK, Abingdon) urine kit. Where required, samples were diluted using cotinine-free serum (fetal calf serum). Absorbance was measured spectrophotometrically at a wavelength of 450 nm. Maternal cotinine levels were categorized into four groups: <70, 70–900, 900–3000 and >3000 ng/ml, which roughly correspond with self-reported non-smoking, 1–4 cigarettes per day, 5–14 cigarettes per day and >14 cigarettes per day, respectively (40).

### Offspring methylation outcome

The main outcome measure in this analysis was DNA methylation level at each of the CpG sites in cord blood samples. However, we also undertook an EWAS for maternal prenatal smoking in samples of peripheral blood when the children were age 7 and 17 years and followed up sites that reached genome-wide significance to investigate the persistence of methylation marks in the offspring over time.

### Confounders

Variables considered as potential confounders in this analysis were maternal age, pre-pregnancy BMI, pre-pregnancy weight, parity, educational attainment, social class, alcohol intake and paternal smoking. Maternal age at delivery was derived from date of birth, which was recorded at that time. At enrolment, the mother was asked to record her height and pre-pregnancy weight, from which BMI was calculated. Mother's parity was also recorded in a questionnaire completed during pregnancy. Based on questionnaire responses, highest educational qualification for the mother was collapsed into one of the five categories from none/ Certificate of Secondary Education (CSE, the lower level of two national school exams that were taken when these women were in school at age 16) to university degree. In addition, the highest parental occupation was used to allocate the children to family social class groups using the 1991 British Office of Population Censuses and Surveys classification. Self-reported alcohol use was obtained in the questionnaire administered at 18 weeks of gestation and individuals were categorized based on whether they were non-drinkers, drank before 18 weeks of gestation or were still drinking alcohol at 18 weeks of gestation. Information on partners’ smoking during pregnancy was obtained from self-reports at 18 weeks of gestation. Where self-reported data on partner smoking were not available (16.3% of partners), maternal reports were used. The bisulphite conversion batch for each sample was also included in the analysis to adjust for batch effects.

### Statistical analysis

Using offspring DNA samples taken from cord blood (at birth), we investigated methylation levels at 466 488 CpG sites across the genome. Methylation *β* values at each CpG site were transformed to obtain *M*-values [log2(*β*/(1 − *β*)] for statistical analysis (62).

Multivariable linear regression was used to perform association tests between maternal cigarette smoking and *M*-values at each CpG site as the outcome. The main exposure measure in our analysis was sustained smoking in pregnancy versus no smoking and the main outcome was cord blood DNA methylation level. Analyses were run with and without adjustment for a number of potential confounders found to be associated with smoking status in pregnancy (Table 2). DNA methylation sites were annotated based on data provided by Illumina (63).

We first identified ‘EWAS-significant’ hits using a Bonferroni correction, where associations below a threshold of 1.07 × 10^−7^ were considered a likely true positive worthy of further examination. However, this Bonferroni correction assumes independent tests and so, as correlation of DNA methylation within gene regions means that CpG sites may not be truly independent, a less conservative FDR procedure based on the Benjamini–Hochberg method was also used to account for multiple testing (65). For this, CpG sites with FDR less than a 0.05 threshold were labelled as EWAS-significant.

It has been demonstrated that differences in methylation can arise as a result of variability of cell composition in whole blood (56). As smoking is known to influence cell composition (66), in order to ensure the results are not influenced by variation in cell-type fraction between samples, we estimated the fraction of CD8T-, CD4T-, NK- and B-cells, monocytes and granulocytes in the samples using the *estimateCellCounts* function in the *minfi* Bioconductor package implemented in R (67). We investigated differences in estimated cell count by smoking status and analyses were repeated adjusting for cell composition by including each blood cell fraction as a covariate in the multivariate linear regression.

Given the previous evidence for sex-specific DNA methylation differences in relation to prenatal smoke exposure (68), we undertook EWAS stratified by sex of the offspring and investigated whether there were any sex-specific CpG sites found to be associated with maternal smoke exposure.

We next used a web-based plotting tool, coMET (44), to investigate the genomic regions of interest from our main EWAS analysis. This tool permits the visualization of methylation correlation between CpG sites, which was limited to a maximum of 75 CpG sites around to the top site of interest and within the gene region identified. In addition, the plots were annotated with functional genomic features based on the ENCODE project (geneENSEMBL, CGI, ChromHMM, DNAse, RegENSEMBL and SNPs).

Further analyses were performed to investigate whether the level of methylation differed depending on the duration and intensity of smoking to which the offspring were exposed *in utero*. For this, the untransformed methylation *β* values for the top CpG sites in each gene region reaching genome-wide significance were plotted against a variable for the duration of smoking in pregnancy as well as the intensity of smoking in pregnancy.

We also investigated whether the methylation alterations associated with prenatal exposure to maternal smoking persisted when the offspring were age 7 and 17 years. Longitudinal methylation data were extracted from each of probe which exceeded the Bonferroni threshold in cord blood. A multilevel model (50,51) including a random intercept and a linear regression spline term to allow for flexibility was fitted to each of these CpG sites sequentially:

$$\begin{array}{cc} \text{met}{\text{h}}_{ij} & =\beta_{0}\;+\;u_{0i}\;+\;\beta_{1}\;\text{sustaine}{\text{d}}_{i}\;+\;\beta_{2}\;\text{ag}{\text{e}}_{ij}\;+\;\beta_{3}\;(\text{ag}{\text{e}}_{ij}-\text{7}{)}_{+\;}\\ \; & \;+\;\beta_{4}\;\text{sustaine}{\text{d}}_{i}\;\text{ag}{\text{e}}_{ij}\;+\;\beta_{5}\;\text{sustaine}{\text{d}}_{i}\;(\text{ag}{\text{e}}_{ij}-\text{7}{)}_{\;+\;}\\ & \;\;+\;\mathrm{confounders}\;+\;\epsilon_{ij} \end{array}$$

$$\epsilon_{ij}\tilde{\;}N(0,\;\sigma_{\epsilon }^{2})$$

$$u_{0i}\tilde{\;}N(0,\;\sigma_{u}^{2})$$

where *i* = 1, … (770) indexes the offspring in the analyses, *j* = 1, 2, 3 indexes the measurement occasion and *a*_+_ = *a* if *a* > 0 or 0 otherwise. *β*_1_ gives the average difference between smoker and non-smoker offspring; *β*_2_ gives the average change in methylation from birth to adolescence; *β*_3_ tells us whether there is any change in this trend (i.e. *β*_2_) from childhood to adolescence; *β*_4_ tells us whether there is a difference in methylation change between smoker and non-smoker offspring and *β*_5_ tells us whether offspring of smokers and non-smokers have a *different* change to the trend (i.e. *β*_2_) of methylation change from birth to childhood. From these we can calculate the change in methylation from 0 to 7 for children of non-smokers (*β*_2_) and smokers (*β*_2_*+ β*_4_), and the change from 7 to 17 for children of non-smokers (*β*_2_ + *β*_3_) and smokers (*β*_2_ + *β*_3_ + *β*_4_ + *β*_5_). For each CpG site, we used a multilevel model, adjusting for batch and the first 20 independent surrogate variable components (which account for heterogeneity between the cord blood and peripheral blood samples).

Strategies were then implemented to estimate the potential role of non-intrauterine mechanisms in the observed associations at later time points. We first considered the potential role of postnatal parental smoking in explaining the persistence of methylation differences at age 7 and both parental and own smoking at age 17 in the offspring of mothers who smoked compared with the offspring of mothers who did not smoke in pregnancy. Additional information about mothers’ smoking status postnatally was obtained in several questionnaires administered after birth, including 8 weeks postpartum and 61 months postpartum. In addition, information about own smoking status was obtained in questionnaires completed by the offspring when they were age 17.

For offspring methylation at age 7, we wished to disentangle a potential causal effect of maternal smoke exposure *in utero* (i.e. a ‘critical period’ hypothesis) from other lifecourse effects, including the existence of postnatal critical periods of maternal smoke exposure, an accumulation of risk with exposure over time or effect modification of *in utero* exposure by postnatal exposure (52). We implemented a structured approach to model the effects of the binary maternal smoking exposure at three time points (in pregnancy and postnatally at 8 weeks and 61 months) on offspring methylation. This involved first fitting a saturated model with one coefficient for each combination of exposures at the three time points (maternal smoking in pregnancy × 8 weeks postnatally × 61 months postnatally) using the lm function in R (version 3.0.1). We then specified a series of nested models corresponding to each lifecourse hypothesis to be tested against the saturated model using an ANOVA test, with a smaller *F*-statistic and a larger *P*-value indicating a better fit of the data to that model. Nested models considered were an *in utero* critical period (maternal smoking in pregnancy), later life critical periods (maternal smoking 8 weeks and maternal smoking 61 months postnatally), accumulation of risk across the three time points (maternal smoking in pregnancy + 8 weeks postnatally + 61 months postnatally) and effect modification of *in utero* exposure postnatally by smoking at the later time points [maternal smoking in pregnancy + (smoking in pregnancy : 8 weeks postnatally) + (maternal smoking in pregnancy : 61 months postnatally)].

For methylation at age 17, we also considered the potential influence of own smoking by the offspring in explaining persistence in methylation signatures associated with intrauterine exposure by running the same multivariable linear regressions this time between own smoking status and methylation as the outcome. From the questionnaires administered when the adolescents were age 17, adolescents who reported that they smoked more than one cigarette per week were classified as smokers and those who said they had never tried a cigarette at either time point were classified as non-smokers and used as the reference category. For this analysis, we did a look-up of the top hits in the maternal smoking analysis in relation to own smoking in order to contrast effect sizes for personal versus maternal smoking associations with methylation at this time point. In addition, we repeated the main analysis at this time point excluding those offspring who reported own smoking to investigate whether this had any influence on the results.

Finally, we compared associations of mothers' and mothers' partners' smoking during pregnancy with offspring methylation at the three time points (birth, age 7 and age 17), using partner smoking during pregnancy as a negative control (34,36–40). Information on partners' smoking status during pregnancy was obtained in a questionnaire administered at 18 weeks of gestation. In addition, mothers were asked about their partner's current smoking at 18 weeks of gestation. The correlation between partner self-report and maternal report of partner smoking was high (*r* = 0.95) and therefore maternal report was used when partners' self-report information was missing. Mutually adjusted models were built by including both maternal and partner smoking to account for potential confounding by the smoking behaviour of the other parent.

EWAS were performed using the ‘CpG assoc’ package (version 2.11) implemented in R (version 3.0.1), multilevel modelling was performed using Stata (version 13) and coMET was run via the web interface (http://epigen.kcl.ac.uk/comet/upload.html). All other analyses were implemented in R (version 3.0.1).

## Supplementary Material

Supplementary Material is available at *HMG* online.

## Funding

This work was supported by the UK Biotechnology and Biological Sciences Research Council (BB/I025751/1 and BB/I025263/1); the UK Medical Research Council and University of Bristol (MC_UU_12013); the Wellcome Trust (WT083431MF to R.C.R.); the Economic and Social Research Council (RES-060–23-0011 to G.D.S. and C.L.R.) and the European Research Council (DEVHEALTH 269874 to G.D.S.). Funding to pay the Open Access publication charges for this article was provided by the University of Bristol RCUK.

## Supplementary Material

Supplementary Data

## References

[1] Bhattacharya S. Campbell D.M. Liston W.A. Bhattacharya S. (2007) Effect of body mass index on pregnancy outcomes in nulliparous women delivering singleton babies. BMC Public Health, 7, 168. 17650297 10.1186/1471-2458-7-168 PMC1940246

[2] Davies D.P. Gray O.P. Ellwood P.C. Abernethy M. (1976) Cigarette-smoking in pregnancy—associations with maternal weight-gain and fetal growth. Lancet, 1, 385–387. 55649 10.1016/s0140-6736(76)90215-4

[3] Sexton M. Hebel J.R. (1984) A clinical-trial of change in maternal smoking and its effect on birth-weight. JAMA, 251, 911–915. 6363731

[4] Kramer M.S. (1987) Determinants of low birth-weight - methodological assessment and meta-analysis. Bull World Health Organ, 65, 663–737. 3322602 PMC2491072

[5] Blake K.V. Gurrin L.C. Evans S.F. Beilin L.J. Landau L.I. Stanley F.J. Newnham J.P. (2000) Maternal cigarette smoking during pregnancy, low birth weight and subsequent blood pressure in early childhood. Early Hum. Dev., 57, 137–147. 10735460 10.1016/s0378-3782(99)00064-x

[6] Lawlor D.A. Najman J.M. Sterne J. Williams G.M. Ebrahim S. Davey Smith G. (2004) Associations of parental, birth, and early life characteristics with systolic blood pressure at 5 years of age—findings from the Mater-University study of pregnancy and its outcomes. Circulation, 110, 2417–2423. 15477400 10.1161/01.CIR.0000145165.80130.B5

[7] von Kries R. Toschke A.M. Koletzko B. Slikker W. (2002) Maternal smoking during pregnancy and childhood obesity. Am. J. Epidemiol., 156, 954–961. 12419768 10.1093/aje/kwf128

[8] Oken E. Levitan E.B. Gillman M.W. (2008) Maternal smoking during pregnancy and child overweight: systematic review and meta-analysis. Int. J. Obes., 32, 201–210. 10.1038/sj.ijo.0803760 PMC2586944 18278059

[9] Ernst M. Moolchan E.T. Robinson M.L. (2001) Behavioral and neural consequences of prenatal exposure to nicotine. J. Am. Acad. Child. Psychiatry, 40, 630–641. 10.1097/00004583-200106000-00007 11392340

[10] Brion M.J. Victora C. Matijasevich A. Horta B. Anselmi L. Steer C. Menezes A.M.B. Lawlor D.A. Davey Smith G. (2010) Maternal smoking and child psychological problems: disentangling causal and noncausal effects. Pediatrics, 126, E57–E65. 20587678 10.1542/peds.2009-2754 PMC3605780

[11] Suter M.A. Anders A.M. Aagaard K.M. (2013) Maternal smoking as a model for environmental epigenetic changes affecting birthweight and fetal programming. Mol. Hum. Reprod., 19, 1–166. 23139402 10.1093/molehr/gas050 PMC3521486

[12] Breitling L.P. Yang R.X. Korn B. Burwinkel B. Brenner H. (2011) Tobacco-smoking-related differential DNA methylation: 27 K discovery and replication. Am. J. Hum. Genet., 88, 450–457. 21457905 10.1016/j.ajhg.2011.03.003 PMC3071918

[13] Monick M.M. Beach S.R.H. Plume J. Sears R. Gerrard M. Brody G.H. Philibert R.A. (2012) Coordinated changes in AHRR methylation in lymphoblasts and pulmonary macrophages from smokers. Am. J. Med. Genet. B, 159B, 141–151. 10.1002/ajmg.b.32021 PMC3318996 22232023

[14] Shenker N.S. Polidoro S. van Veldhoven K. Sacerdote C. Ricceri F. Birrell M.A. Belvisi M.G. Brown R. Vineis P. Flanagan J.M. (2013) Epigenome-wide association study in the European Prospective Investigation into Cancer and Nutrition (EPIC-Turin) identifies novel genetic loci associated with smoking. Hum. Mol. Genet., 22, 843–851. 23175441 10.1093/hmg/dds488

[15] Zeilinger S. Kuhnel B. Klopp N. Baurecht H. Kleinschmidt A. Gieger C. Weidinger S. Lattka E. Adamski J. Peters A. (2013) Tobacco smoking leads to extensive genome-wide changes in DNA methylation. PLoS ONE, 8, e63812. 23691101 10.1371/journal.pone.0063812 PMC3656907

[16] Elliott H.R. Tillin T. McArdle W.L. Ho K. Duggirala A. Frayling T.M. Davey Smith G. Hughes A.D. Chaturvedi N. Relton C.L. (2014) Differences in smoking associated DNA methylation patterns in South Asians and Europeans. Clin. Epigenet., 6, 4. 10.1186/1868-7083-6-4 PMC3915234 24485148

[17] Besingi W. Johansson A. (2014) Smoke-related DNA methylation changes in the etiology of human disease. Hum. Mol. Genet., 23, 2290–2297. 24334605 10.1093/hmg/ddt621

[18] Joubert B.R. Haberg S.E. Nilsen R.M. Wang X.T. Vollset S.E. Murphy S.K. Huang Z. Hoyo C. Midttun O. Cupul-Uicab L.A. (2012) 450K epigenome-wide scan identifies differential DNA methylation in newborns related to maternal smoking during pregnancy. Environ. Health Persp., 120, 1425–1431. 10.1289/ehp.1205412 PMC3491949 22851337

[19] Markunas C.A. Xu Z. Harlid S. Wade P.A. Lie R.T. Taylor J.A. Wilcox A.J. (2014) Identification of DNA methylation changes in newborns related to maternal smoking during pregnancy. Environ. Health Perspect. doi:10.1289/ehp.1307892. 10.1289/ehp.1307892 PMC4181928 24906187

[20] Joubert B.R. Haberg S.E. Bell D.A. Nilsen R.M. Vollset S.E. Midttun O. Ueland P.M. Wu M.C. Nystad W. Peddada S.D. (2014) Maternal smoking and DNA methylation in newborns: in utero effect or epigenetic inheritance? Cancer Epidemiol. Biomarkers Prev., 23, 1007–1017. 24740201 10.1158/1055-9965.EPI-13-1256 PMC4140220

[21] Faulk C. Dolinoy D.C. (2011) Timing is everything: the when and how of environmentally induced changes in the epigenome of animals. Epigenetics, 6, 791–797. 21636976 10.4161/epi.6.7.16209 PMC3230539

[22] Lee K.W. Pausova Z. (2013) Cigarette smoking and DNA methylation. Front Genet., 4, 132. 23882278 10.3389/fgene.2013.00132 PMC3713237

[23] Novakovic B. Ryan J. Pereira N. Boughton B. Craig J.M. Saffery R. (2014) Postnatal stability, tissue, and time specific effects of AHRR methylation change in response to maternal smoking in pregnancy. Epigenetics, 9, 377–386. 24270552 10.4161/epi.27248 PMC4053456

[24] Wan E.S. Qiu W. Baccarelli A. Carey V.J. Bacherman H. Rennard S.I. Agusti A. Anderson W. Lomas D.A. Demeo D.L. (2012) Cigarette smoking behaviors and time since quitting are associated with differential DNA methylation across the human genome. Hum. Mol. Genet., 21, 3073–3082. 22492999 10.1093/hmg/dds135 PMC3373248

[25] Breton C.V. Siegmund K.D. Joubert B.R. Wang X. Qui W. Carey V. Nystad W. Haberg S.E. Ober C. Nicolae D. (2014) Prenatal tobacco smoke exposure is associated with childhood DNA CpG methylation. PLoS ONE, 9, e99716. 24964093 10.1371/journal.pone.0099716 PMC4070909

[26] Lee K.W. Richmond R. Hu P. French L. Shin J. Bourdon C. Reischl E. Waldenberger M. Zeilinger S. Gaunt T. (2014) Prenatal exposure to maternal cigarette smoking and DNA methylation: epigenome-wide association in a discovery sample of adolescents and replication in an independent cohort at birth through 17 years of age. Environ. Health Perspect. doi:10.1289/ehp.1408614. 10.1289/ehp.1408614 PMC4314251 25325234

[27] Richiardi L. Bellocco R. Zugna D. (2013) Mediation analysis in epidemiology: methods, interpretation and bias. Int. J. Epidemiol., 42, 1511–1519. 24019424 10.1093/ije/dyt127

[28] Fewell Z. Davey Smith G. Sterne J.A. (2007) The impact of residual and unmeasured confounding in epidemiologic studies: a simulation study. Am. J. Epidemiol., 166, 646–655. 17615092 10.1093/aje/kwm165

[29] Kuh D. Ben-Shlomo Y. Lynch J. Hallqvist J. Power C. (2003) Life course epidemiology. J. Epidemiol. Community Health, 57, 778–783. 14573579 10.1136/jech.57.10.778 PMC1732305

[30] Philibert R.A. Beach S.R. Brody G.H. (2012) Demethylation of the aryl hydrocarbon receptor repressor as a biomarker for nascent smokers. Epigenetics, 7, 1331–1338. 23070629 10.4161/epi.22520 PMC3499333

[31] Davey Smith G. Lawlor D.A. Harbord R. Timpson N. Day I. Ebrahim S. (2007) Clustered environments and randomized genes: a fundamental distinction between conventional and genetic epidemiology. PLoS Med., 4, e352. 18076282 10.1371/journal.pmed.0040352 PMC2121108

[32] Davey Smith G. (2012) Epigenesis for epidemiologists: does evo-devo have implications for population health research and practice? Int. J. Epidemiol., 41, 236–247. 22422459 10.1093/ije/dys016

[33] Relton C.L. Davey Smith G. (2010) Epigenetic epidemiology of common complex disease: prospects for prediction, prevention, and treatment. PLoS Med., 7, e1000356. 21048988 10.1371/journal.pmed.1000356 PMC2964338

[34] Davey Smith G. (2008) Assessing intrauterine influences on offspring health outcomes: can epidemiological findings yield robust results? (vol 102, pg 245, 2008). Basic Clin. Pharmacol. Toxicol., 102, 489. 10.1111/j.1742-7843.2007.00191.x 18226080

[35] Relton C.L. Davey Smith G. (2012) Two-step epigenetic Mendelian randomization: a strategy for establishing the causal role of epigenetic processes in pathways to disease. Int. J. Epidemiol., 41, 161–176. 22422451 10.1093/ije/dyr233 PMC3304531

[36] Brion M.J.A. Leary S.D. Smith G.D. Ness A.R. (2007) Similar associations of parental prenatal smoking suggest child blood pressure is not influenced by intrauterine effects. Hypertension, 49, 1422–1428. 17404184 10.1161/HYPERTENSIONAHA.106.085316

[37] Langley K. Heron J. Smith G.D. Thapar A. (2012) Maternal and paternal smoking during pregnancy and risk of ADHD symptoms in offspring: testing for intrauterine effects. Am. J. Epidemiol., 176, 261–268. 22791738 10.1093/aje/kwr510 PMC3406617

[38] Howe L.D. Matijasevich A. Tilling K. Brion M.J. Leary S.D. Davey Smith G. Lawlor D.A. (2012) Maternal smoking during pregnancy and offspring trajectories of height and adiposity: comparing maternal and paternal associations. Int. J. Epidemiol., 41, 722–732. 22407859 10.1093/ije/dys025 PMC3396309

[39] Taylor A.E. Howe L.D. Heron J.E. Ware J.J. Hickman M. Munafo M.R. (2014) Maternal smoking during pregnancy and offspring smoking initiation: assessing the role of intrauterine exposure. Addiction, 109, 1013–1021. 24521169 10.1111/add.12514 PMC4114534

[40] Taylor A.E. Davey Smith G. Bares C.B. Edwards A.C. Munafo M.R. (2014) Partner smoking and maternal cotinine during pregnancy: implications for negative control methods. Drug Alcohol. Depend., 139, 159–163. 24726428 10.1016/j.drugalcdep.2014.03.012 PMC4026952

[41] Boyd A. Golding J. Macleod J. Lawlor D.A. Fraser A. Henderson J. Molloy L. Ness A. Ring S. Smith G.D. (2013) Cohort profile: the ‘Children of the 90s’—the index offspring of the Avon Longitudinal Study of Parents and Children. Int. J. Epidemiol., 42, 111–127. 22507743 10.1093/ije/dys064 PMC3600618

[42] Fraser A. Macdonald-Wallis C. Tilling K. Boyd A. Golding J. Smith G.D. Henderson J. Macleod J. Molloy L. Ness A. (2013) Cohort profile: the Avon Longitudinal Study of Parents and Children: ALSPAC mothers cohort. Int. J. Epidemiol., 42, 97–110. 22507742 10.1093/ije/dys066 PMC3600619

[43] Naeem H. Wong N.C. Chatterton Z. Hong M.K. Pedersen J.S. Corcoran N.M. Hovens C.M. Macintyre G. (2014) Reducing the risk of false discovery enabling identification of biologically significant genome-wide methylation status using the HumanMethylation450 array. BMC Genomics, 15, 51. 24447442 10.1186/1471-2164-15-51 PMC3943510

[44] Martin T.C. Yet I. Tsai P. Bell J.T. (2014) coMET: visualisation of regional epigenome-wide association scan (EWAS) results and DNA co-methylation patterns http://epigen.kcl.ac.uk/comet.

[45] Yuen R.K. Neumann S.M. Fok A.K. Penaherrera M.S. McFadden D.E. Robinson W.P. Kobor M.S. (2011) Extensive epigenetic reprogramming in human somatic tissues between fetus and adult. Epigenet Chromatin, 4, 7. 10.1186/1756-8935-4-7 PMC3112062 21545704

[46] Davey Smith G. Ebrahim S. (2003) ‘Mendelian randomization’: can genetic epidemiology contribute to understanding environmental determinants of disease? Int. J. Epidemiol., 32, 1–22. 12689998 10.1093/ije/dyg070

[47] Davey Smith G. Hemani G. (2014) Mendelian randomization: genetic anchors for causal inference in epidemiological studies. Hum. Mol. Genet., 23, R89–R98. 25064373 10.1093/hmg/ddu328 PMC4170722

[48] Ware J.J. van den Bree M.B. Munafo M.R. (2011) Association of the CHRNA5-A3-B4 gene cluster with heaviness of smoking: a meta-analysis. Nicotine Tob. Res., 13, 1167–1175. 22071378 10.1093/ntr/ntr118 PMC3223575

[49] Freathy R.M. Ring S.M. Shields B. Galobardes B. Knight B. Weedon M.N. Davey Smith G. Frayling T.M. Hattersley A.T. (2009) A common genetic variant in the 15q24 nicotinic acetylcholine receptor gene cluster (CHRNA5-CHRNA3-CHRNB4) is associated with a reduced ability of women to quit smoking in pregnancy. Hum. Mol. Genet., 18, 2922–2927. 19429911 10.1093/hmg/ddp216 PMC2706684

[50] Laird N.M. Ware J.H. (1982) Random-effects models for longitudinal data. Biometrics, 38, 963–974. 7168798

[51] Goldstein H. (1986) Multilevel mixed linear-model analysis using iterative generalized least-squares. Biometrika, 73, 43–56.

[52] Mishra G. Nitsch D. Black S. De Stavola B. Kuh D. Hardy R. (2009) A structured approach to modelling the effects of binary exposure variables over the life course. Int. J. Epidemiol., 38, 528–537. 19028777 10.1093/ije/dyn229 PMC2663717

[53] Pembrey M.E. Bygren L.O. Kaati G. Edvinsson S. Northstone K. Sjostrom M. Golding J. Team A.S. (2006) Sex-specific, male-line transgenerational responses in humans. Eur. J. Hum. Genet., 14, 159–166. 16391557 10.1038/sj.ejhg.5201538

[54] Soubry A. Schildkraut J.M. Murtha A. Wang F. Huang Z.Q. Bernal A. Kurtzberg J. Jirtle R.L. Murphy S.K. Hoyo C. (2013) Paternal obesity is associated with IGF2 hypomethylation in newborns: results from a Newborn Epigenetics Study (NEST) cohort. BMC Med., 11, 29. 23388414 10.1186/1741-7015-11-29 PMC3584733

[55] Northstone K. Golding J. Davey Smith G. Miller L.L. Pembrey M. (2014) Prepubertal start of father's smoking and increased body fat in his sons: further characterisation of paternal transgenerational responses. Eur. J. Hum. Genet., 22, 1382–1386. 24690679 10.1038/ejhg.2014.31 PMC4085023

[56] Reinius L.E. Acevedo N. Joerink M. Pershagen G. Dahlen S.E. Greco D. Soderhall C. Scheynius A. Kere J. (2012) Differential DNA methylation in purified human blood cells: implications for cell lineage and studies on disease susceptibility. PLoS ONE, 7, e41361. 22848472 10.1371/journal.pone.0041361 PMC3405143

[57] Hogg K. Price E.M. Hanna C.W. Robinson W.P. (2012) Prenatal and perinatal environmental influences on the human fetal and placental epigenome. Clin. Pharmacol. Ther., 92, 716–726. 23047650 10.1038/clpt.2012.141

[58] Waterland R.A. Michels K.B. (2007) Epigenetic epidemiology of the developmental origins hypothesis. Annu. Rev. Nutr., 27, 363–388. 17465856 10.1146/annurev.nutr.27.061406.093705

[59] Reynolds R.M. Jacobsen G.H. Drake A.J. (2013) What is the evidence in humans that DNA methylation changes link events in utero and later life disease? Clin*.* Endocrinol., 78, 814–822. 10.1111/cen.12164 23374091

[60] Kirkbride J.B. Susser E. Kundakovic M. Kresovich J.K. Davey Smith G. Relton C.L. (2012) Prenatal nutrition, epigenetics and schizophrenia risk: can we test causal effects? Epigenomics, 4, 303–315. 22690666 10.2217/epi.12.20 PMC3970193

[61] Dedeurwaerder S. Defrance M. Calonne E. Denis H. Sotiriou C. Fuks F. (2011) Evaluation of the infinium methylation 450 K technology. Epigenomics, 3, 771–784. 22126295 10.2217/epi.11.105

[62] Du P. Zhang X. Huang C.C. Jafari N. Kibbe W.A. Hou L. Lin S.M. (2010) Comparison of Beta-value and M-value methods for quantifying methylation levels by microarray analysis. BMC Bioinformatics, 11, 587. 21118553 10.1186/1471-2105-11-587 PMC3012676

[63] Bibikova M. Barnes B. Tsan C. Ho V. Klotzle B. Le J.M. Delano D. Zhang L. Schroth G.P. Gunderson K.L. (2011) High density DNA methylation array with single CpG site resolution. Genomics, 98, 288–295. 21839163 10.1016/j.ygeno.2011.07.007

[64] Touleimat N. Tost J. (2012) Complete pipeline for Infinium((R)) Human Methylation 450 K BeadChip data processing using subset quantile normalization for accurate DNA methylation estimation. Epigenomics, 4, 325–341. 22690668 10.2217/epi.12.21

[65] Benjamini Y. Drai D. Elmer G. Kafkafi N. Golani I. (2001) Controlling the false discovery rate in behavior genetics research. Behav. Brain. Res., 125, 279–284. 11682119 10.1016/s0166-4328(01)00297-2

[66] Sunyer J. Munoz A. Peng Y. Margolick J. Chmiel J.S. Oishi J. Kingsley L. Samet J.M. (1996) Longitudinal relation between smoking and white blood cells. Am. J. Epidemiol., 144, 734–741. 8857822 10.1093/oxfordjournals.aje.a008997

[67] Jaffe A.E. Irizarry R.A. (2014) Accounting for cellular heterogeneity is critical in epigenome-wide association studies. Genome Biology, 15, R31. 24495553 10.1186/gb-2014-15-2-r31 PMC4053810

[68] Murphy S.K. Adigun A. Huang Z. Overcash F. Wang F. Jirtle R.L. Schildkraut J.M. Murtha A.P. Iversen E.S. Hoyo C. (2012) Gender-specific methylation differences in relation to prenatal exposure to cigarette smoke. Gene, 494, 36–43. 22202639 10.1016/j.gene.2011.11.062 PMC3627389

