# Supplementary material for: Prenatal exposure to maternal smoking and offspring DNA methylation across the lifecourse: findings from the Avon Longitudinal Study of Parents and Children (ALSPAC)
Source: Hum Mol Genet. 2014 Dec 30;24(8):2201–17. doi: 10.1093/hmg/ddu739 (PMC4380069; doi:10.1093/hmg/ddu739)
Supplement: Supplementary Data [file supp_24_8_2201__index.html]

Prenatal exposure to maternal smoking and offspring DNA methylation across the lifecourse: Findings from the Avon Longitudinal Study of Parents and Children (ALSPAC) — Prenatal exposure to maternal smoking and offspring DNA methylation across the lifecourse: findings from the Avon Longitudinal Study of Parents and Children (ALSPAC) — Prenatal exposure to maternal smoking and offspring DNA methylation across the lifecourse: findings from the Avon Longitudinal Study of Parents and Children (ALSPAC) — Supplementary Data 

# Prenatal exposure to maternal smoking and offspring DNA methylation across the lifecourse: findings from the Avon Longitudinal Study of Parents and Children (ALSPAC)

## Supplementary Data

Supplementary Data

**Files in this Data Supplement:**

- Supplementary FigureS1-S7 - pdf file
- Supplementary FigureS8 - pdf file
- Supplementary Data - Docx file
- Supplementary Tables - xlsx file
