# Supplementary material for: Prenatal exposure to maternal smoking and offspring DNA methylation across the lifecourse: findings from the Avon Longitudinal Study of Parents and Children (ALSPAC)
Source: Hum Mol Genet. 2014 Dec 30;24(8):2201–17. doi: 10.1093/hmg/ddu739 (PMC4380069; doi:10.1093/hmg/ddu739)
Supplement: Supplementary Data [file supp_ddu739_ddu739supp_figS1-S7.pdf]

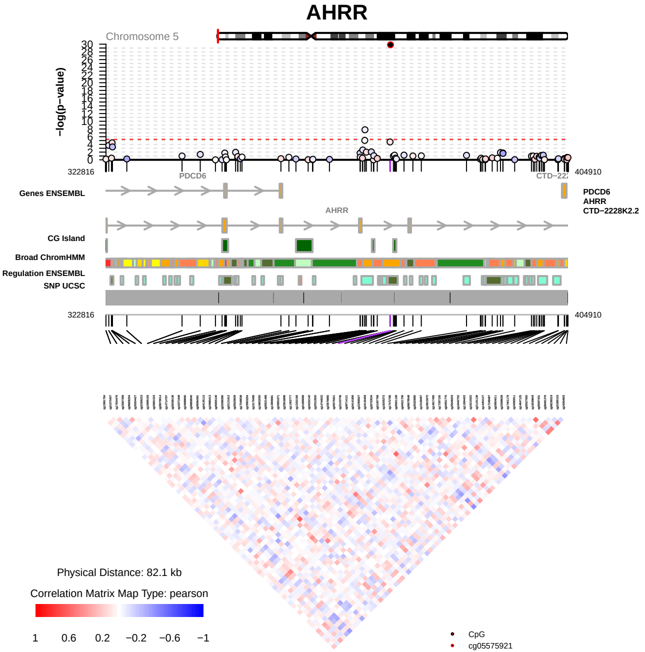
AHRR
Chromosome 5
−log(p−value)
30
28
26
24
22
20
18
16
14
12
10
8
6
4
2
0
322816
404910
PDCD6
CTD−22
Genes ENSEMBL
AHRR
PDCD6
AHRR
CTD−2228K2.2
CG Island
Broad ChromHMM
Regulation ENSEMBL
SNP UCSC
322816
404910
Physical Distance: 82.1 kb
Correlation Matrix Map Type: pearson
1
0.6
0.2
−0.2
−0.6
−1
CpG
cg05575921

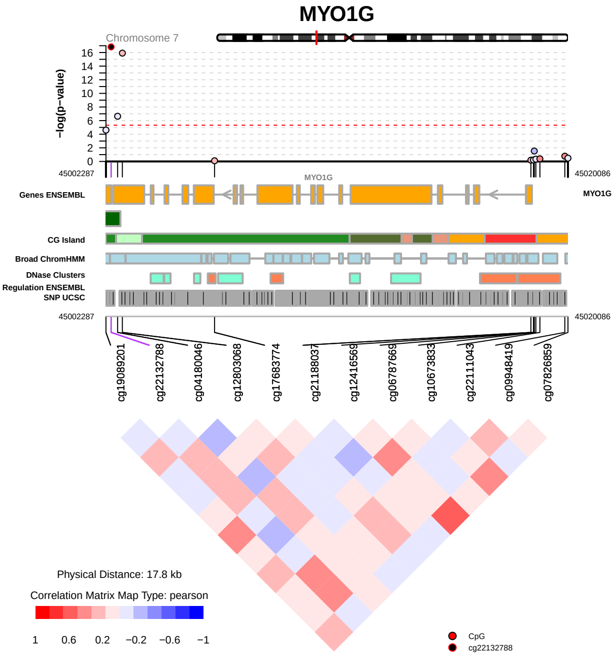

MYO1G
Chromosome 7
−log(p−value)
16
14
12
10
8
6
4
2
0
45002287
45020086
MYO1G
Genes ENSEMBL
MYO1G
CG Island
Broad ChromHMM
DNase Clusters
Regulation ENSEMBL
SNP UCSC
45002287
45020086
cg19089201
cg22132788
cg04180046
cg12803068
cg17683774
cg21188037
cg12416569
cg06787669
cg10673833
cg22111043
cg09948419
cg07826859
Physical Distance: 17.8 kb
Correlation Matrix Map Type: pearson
1
0.6
0.2
−0.2
−0.6
−1
CpG
cg22132788

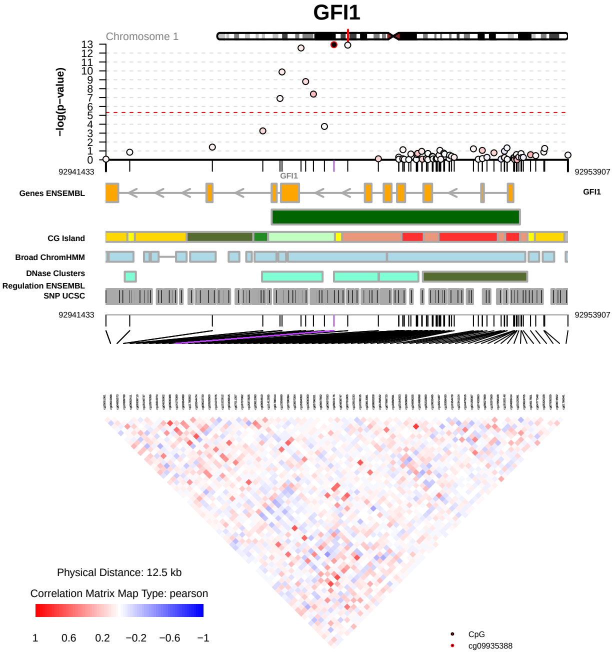
GFI1
Chromosome 1
−log(p−value)
13
12
11
10
9
8
7
6
5
4
3
2
1
0
92941433
92953907
GFI1
Genes ENSEMBL
GFI1
CG Island
Broad ChromHMM
DNase Clusters
Regulation ENSEMBL
SNP UCSC
92941433
92953907
Physical Distance: 12.5 kb
Correlation Matrix Map Type: pearson
1
0.6
0.2
−0.2
−0.6
−1
CpG
cg09935388

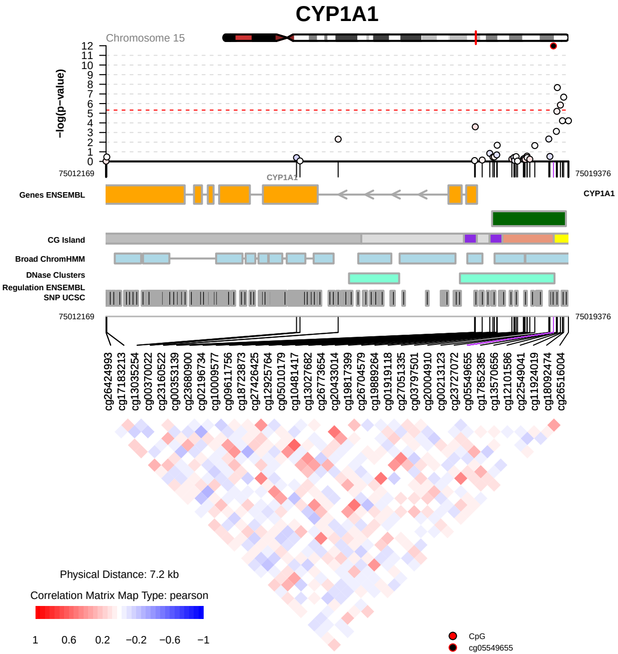

CYP1A1
Chromosome 15
−log(p−value)
12
11
10
9
8
7
6
5
4
3
2
1
0
75012169
75019376
CYP1A1
Genes ENSEMBL
CYP1A1
CG Island
Broad ChromHMM
DNase Clusters
Regulation ENSEMBL
SNP UCSC
75012169
75019376
cg26424993
cg17183213
cg13035254
cg00370022
cg23160522
cg00353139
cg23680900
cg02196734
cg10009577
cg09611756
cg18723873
cg27426425
cg12925764
cg05010179
cg10481417
cg13027682
cg26773654
cg20433014
cg19817399
cg26704579
cg19889264
cg01919118
cg27051335
cg03797501
cg20004910
cg00213123
cg23727072
cg05549655
cg17852385
cg13570656
cg12101586
cg22549041
cg11924019
cg18092474
cg26516004
Physical Distance: 7.2 kb
Correlation Matrix Map Type: pearson
1
0.6
0.2
−0.2
−0.6
−1
CpG
cg05549655

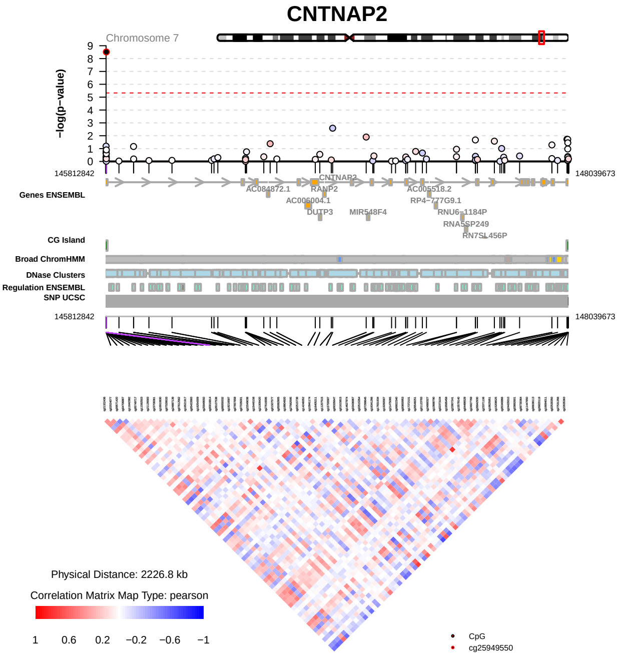

CNTNAP2
Chromosome 7
−log(p−value)
9
8
7
6
5
4
3
2
1
0
145812842
148039673
CNTNAP2
AC084872.1
RANP2
AC006004.1
DUTP3
MIR548F4
AC005518.2
RP4−777G9.1
RNU6−1184P
RNA5SP249
RN7SL456P
Genes ENSEMBL
CG Island
Broad ChromHMM
DNase Clusters
Regulation ENSEMBL
SNP UCSC
145812842
148039673
Physical Distance: 2226.8 kb
Correlation Matrix Map Type: pearson
1
0.6
0.2
−0.2
−0.6
−1
CpG
cg25949550

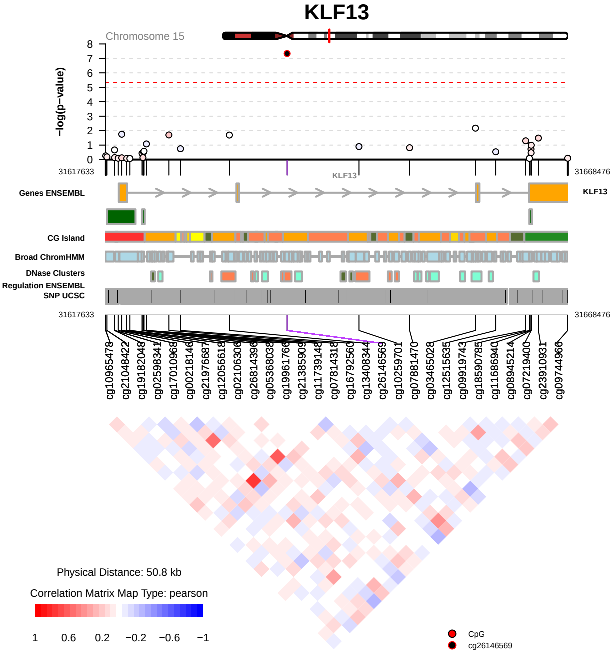
KLF13
Chromosome 15
−log(p−value)
8
7
6
5
4
3
2
1
0
31617633
31668476
KLF13
Genes ENSEMBL
KLF13
CG Island
Broad ChromHMM
DNase Clusters
Regulation ENSEMBL
SNP UCSC
31617633
31668476
cg10965478
cg21048422
cg19182048
cg02598341
cg17010968
cg00218146
cg21976687
cg12056618
cg02106306
cg26814396
cg05368038
cg19961768
cg21385909
cg11739148
cg07814318
cg16792560
cg13408344
cg26146569
cg10259701
cg07881470
cg03465028
cg12515635
cg09919743
cg18590785
cg11686940
cg08945214
cg07219400
cg23910931
cg09744966
Physical Distance: 50.8 kb
Correlation Matrix Map Type: pearson
1
0.6
0.2
−0.2
−0.6
−1
CpG
cg26146569

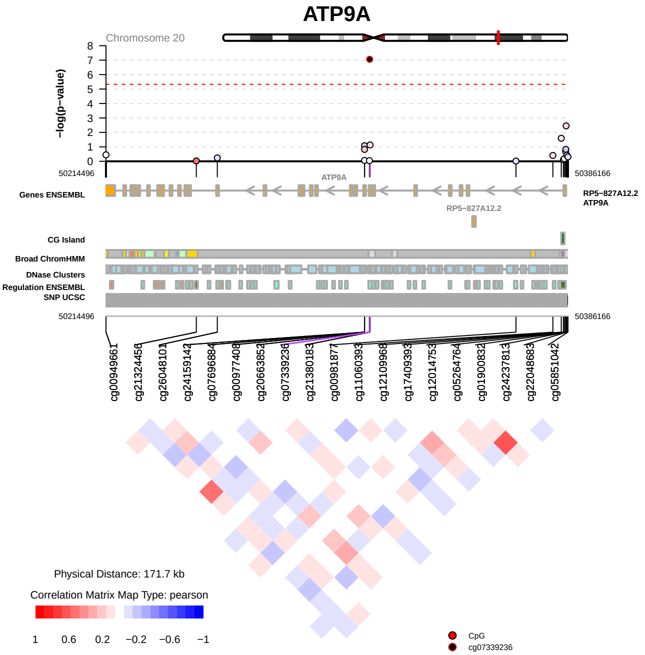
ATP9A
Chromosome 20
−log(p−value)
8
7
6
5
4
3
2
1
0
50214496
50386166
ATP9A
Genes ENSEMBL
RP5−827A12.2
ATP9A
RP5−827A12.2
CG Island
Broad ChromHMM
DNase Clusters
Regulation ENSEMBL
SNP UCSC
50214496
50386166
cg00949661
cg21324456
cg26048101
cg24159142
cg07696884
cg00977408
cg20663852
cg07339236
cg21380183
cg00981877
cg11060393
cg12109968
cg17409393
cg12014753
cg05264764
cg01900832
cg24237813
cg22048683
cg05851042
Physical Distance: 171.7 kb
Correlation Matrix Map Type: pearson
1
0.6
0.2
−0.2
−0.6
−1
CpG
cg07339236
