# Supplementary figures and images for: Prenatal exposure to maternal smoking and offspring DNA methylation across the lifecourse: findings from the Avon Longitudinal Study of Parents and Children (ALSPAC)
Source: Hum Mol Genet. 2014 Dec 30;24(8):2201–17. doi: 10.1093/hmg/ddu739 (PMC4380069; doi:10.1093/hmg/ddu739)

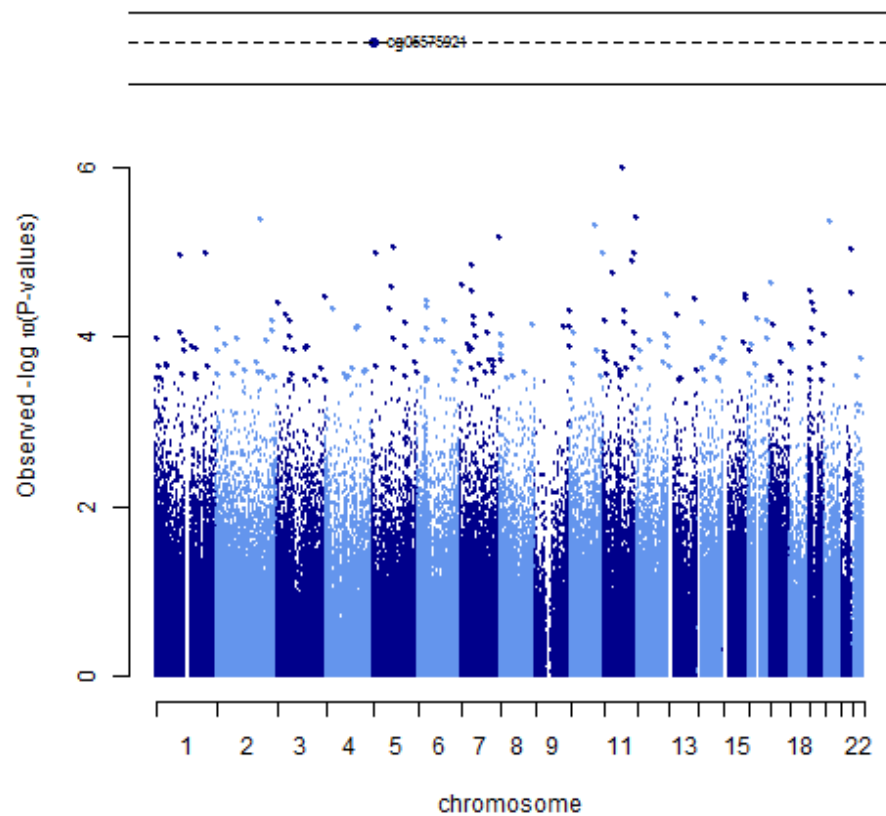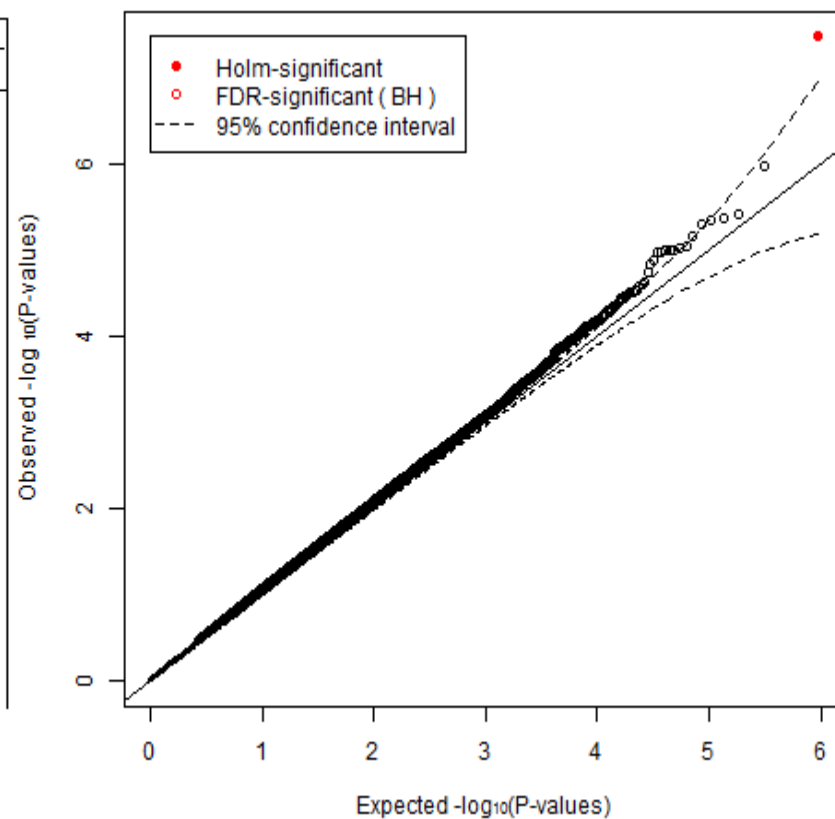

Supplement: Supplementary Data [file supp_ddu739_ddu739supp_figS8.pdf]
