# Supplementary material for: Prenatal exposure to maternal smoking and offspring DNA methylation across the lifecourse: findings from the Avon Longitudinal Study of Parents and Children (ALSPAC)
Source: Hum Mol Genet. 2014 Dec 30;24(8):2201–17. doi: 10.1093/hmg/ddu739 (PMC4380069; doi:10.1093/hmg/ddu739)
Supplement: Supplementary Data [file supp_ddu739_ddu739supp.docx]

Supporting information

Table S1 - Estimated cell type proportions in cord blood of individuals whose mothers did and did not smoke during pregnancy

Table S2 - Differential methylation in cord blood DNA for the offspring of mothers with sustained smoking in pregnancy compared with non-smokers, with cell type correction

Table S3 - Sex-specific associations for differential methylation in cord blood DNA for the offspring of mothers with sustained smoking in pregnancy compared with non-smokers

Table S4 - Change in methylation between 0-7 and 7-17 years for the offspring of sustained smokers in pregnancy and the offspring of non-smokers at the top CpG sites associated with sustained smoking identified in cord blood (N=770)*

Table S5 - Differential methylation in peripheral blood DNA at age 17 for offspring who smoked compared with non-smokers at CpG sites found to be strongly associated with smoking in pregnancy at the same time point

Table S6 - Differential methylation in peripheral blood DNA at age 17 for the offspring of mothers with sustained smoking in pregnancy compared with non-smokers, excluding offspring who also smoked at age 17.

Table S7 - Associations between parental smoking in pregnancy and offspring cord blood DNA methylation at top CpG sites

Table S8 - Associations between parental smoking in pregnancy and offspring age 7 peripheral blood DNA methylation at top CpG sites

Table S9 - Associations between parental smoking in pregnancy and offspring age 17 peripheral blood DNA methylation at top CpG sites

Figures S1 – S7 - Visualization of genomic regions of interest obtained from EWAS using coMET

Figure S8 - Manhattan and QQ plot for epigenome-wide association study of maternal cotinine on cord blood DNA methylation*

*Maternal cotinine levels were categorized into four groups: <70, 70-900, 900-3000 and >3000ng/ml and results were adjusted for batch; N= 374
